# Supplementary figures and images for: The muscle specific MEF2Dα2 isoform promotes muscle ketolysis and running capacity in mice
Source: EMBO Rep. 2025 Sep 16;26(21):5216–38. doi: 10.1038/s44319-025-00578-3 (PMC12592725; doi:10.1038/s44319-025-00578-3)

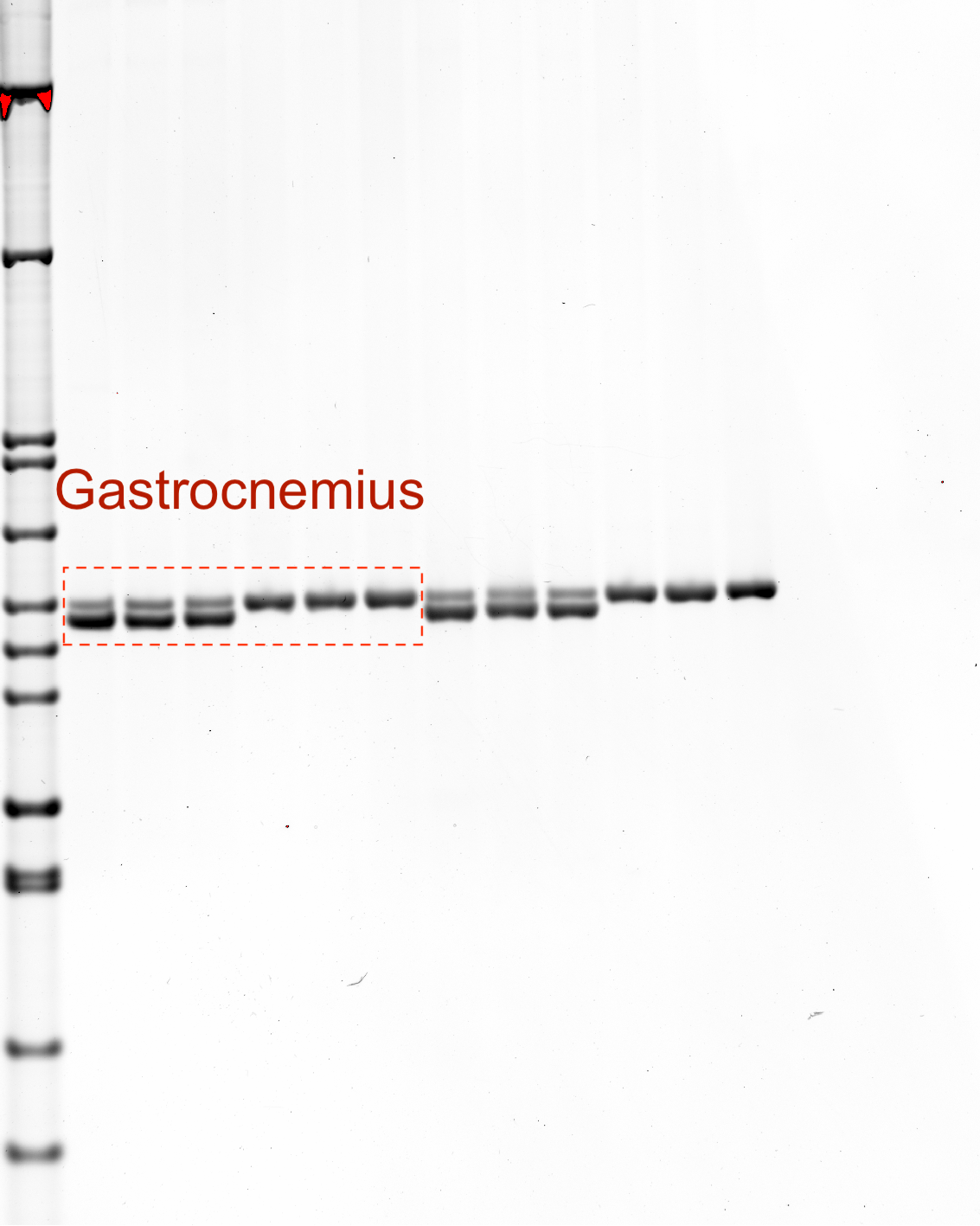

Supplement: Supplementary file 5 — Source data Fig. 1 [file 44319_2025_578_MOESM5_ESM.zip › Figure 1/1A/1A (MEF2D a exon).tif]

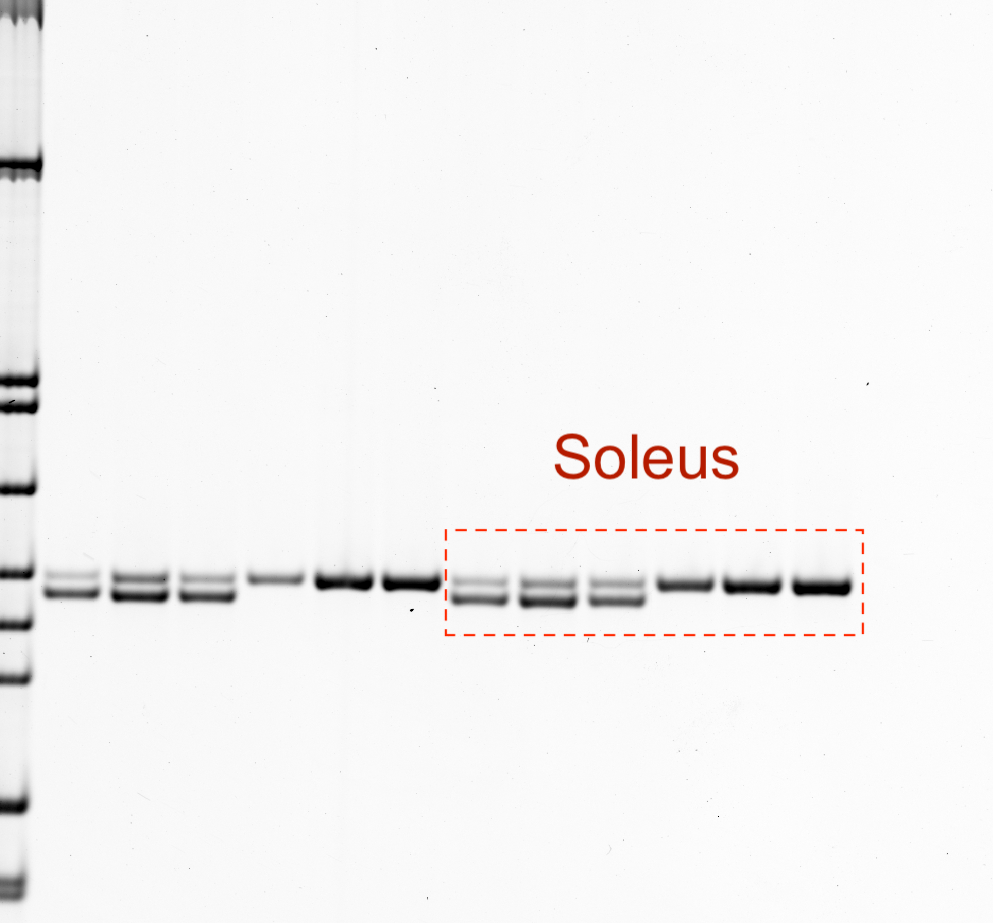

Supplement: Supplementary file 5 — Source data Fig. 1 [file 44319_2025_578_MOESM5_ESM.zip › Figure 1/1A/1A (sol mef2d alpha).tif]

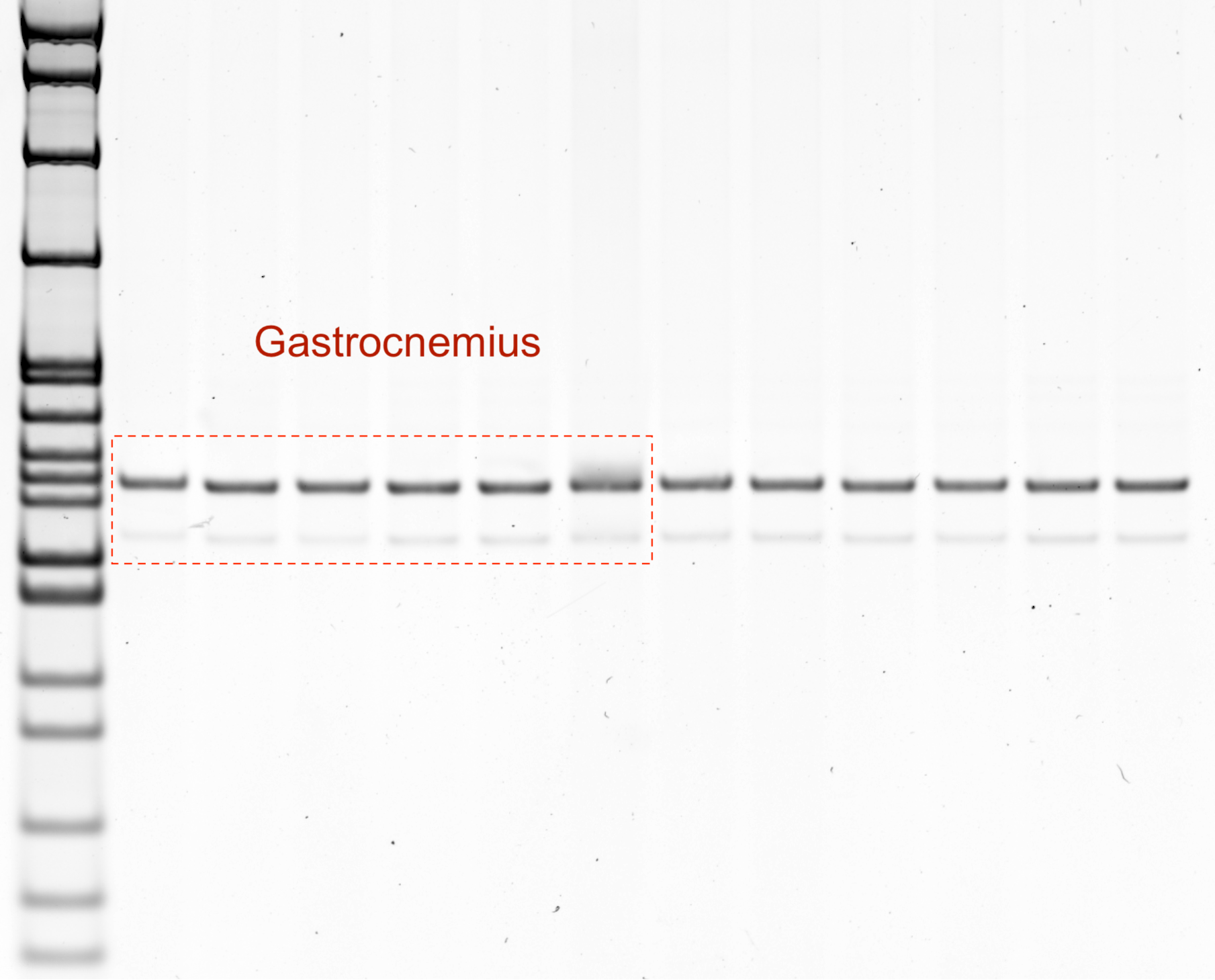

Supplement: Supplementary file 5 — Source data Fig. 1 [file 44319_2025_578_MOESM5_ESM.zip › Figure 1/1A/1A (Mef2d b exon).tif]

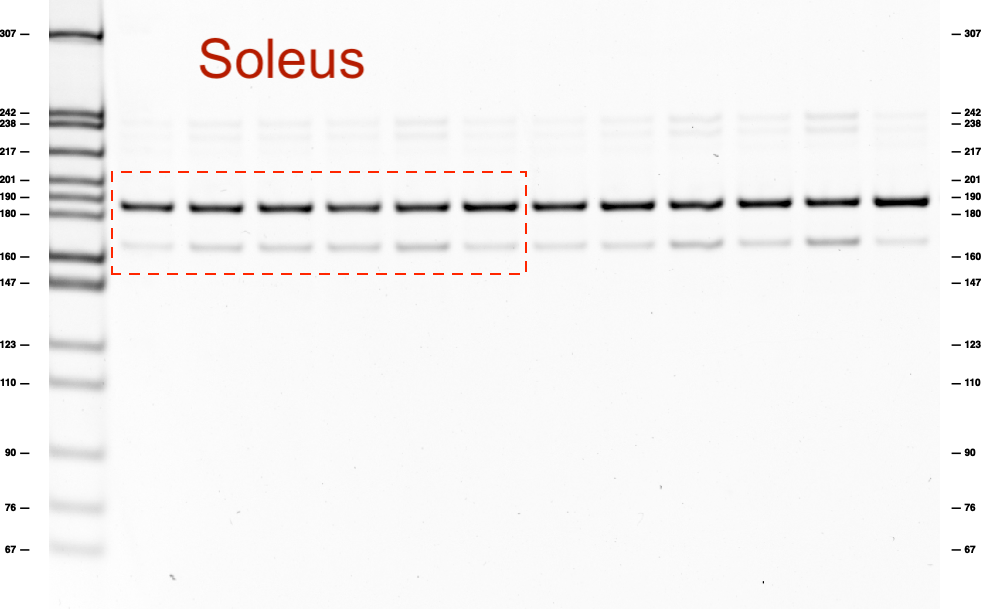

Supplement: Supplementary file 5 — Source data Fig. 1 [file 44319_2025_578_MOESM5_ESM.zip › Figure 1/1A/1A (sol mef2d beta).tif]

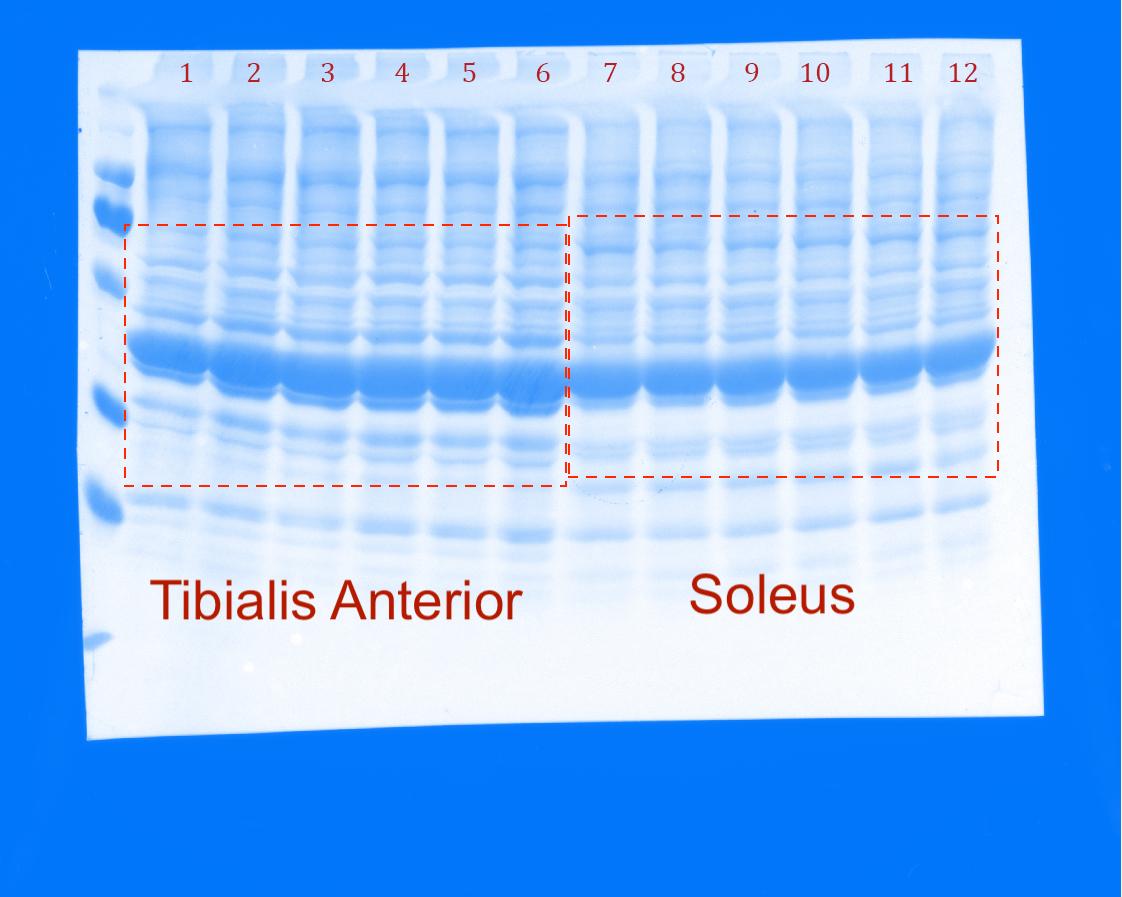

Supplement: Supplementary file 5 — Source data Fig. 1 [file 44319_2025_578_MOESM5_ESM.zip › Figure 1/1B/1B (Coomasie stained MEF2D blot).tif]

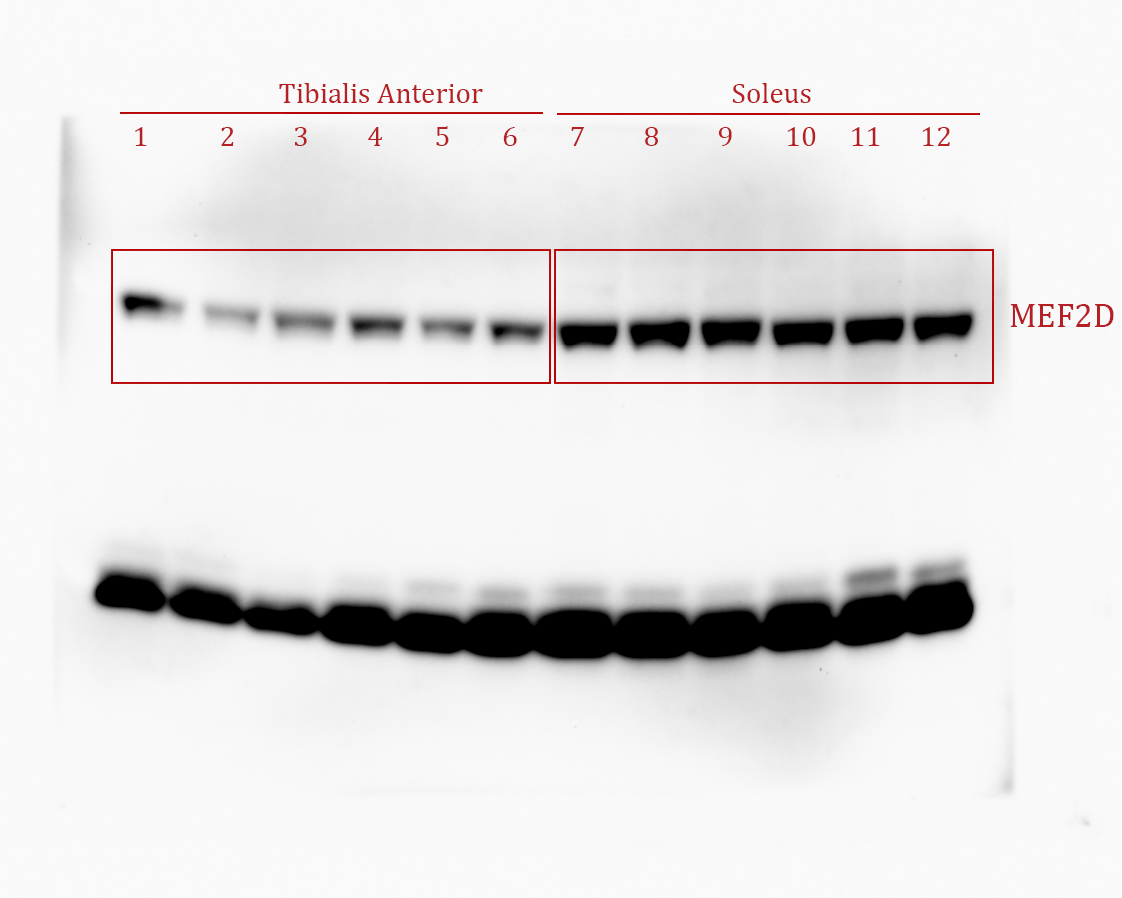

Supplement: Supplementary file 5 — Source data Fig. 1 [file 44319_2025_578_MOESM5_ESM.zip › Figure 1/1B/1B (MEF2D Western blot).tif]

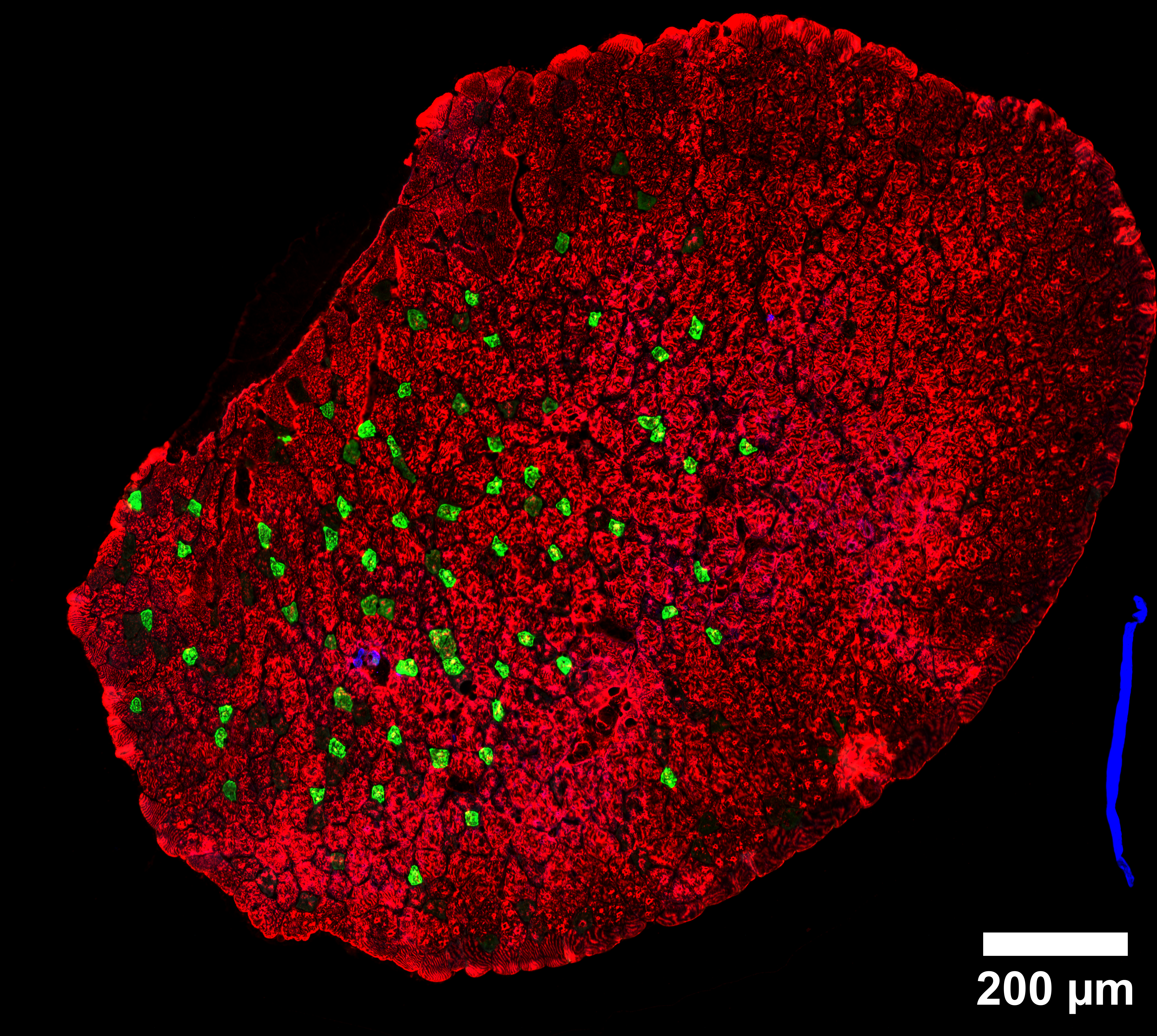

Supplement: Supplementary file 6 — Source data Fig. 2 [file 44319_2025_578_MOESM6_ESM.zip › Figure 2/2D/1047-EDL-EKO-Merged.tif]

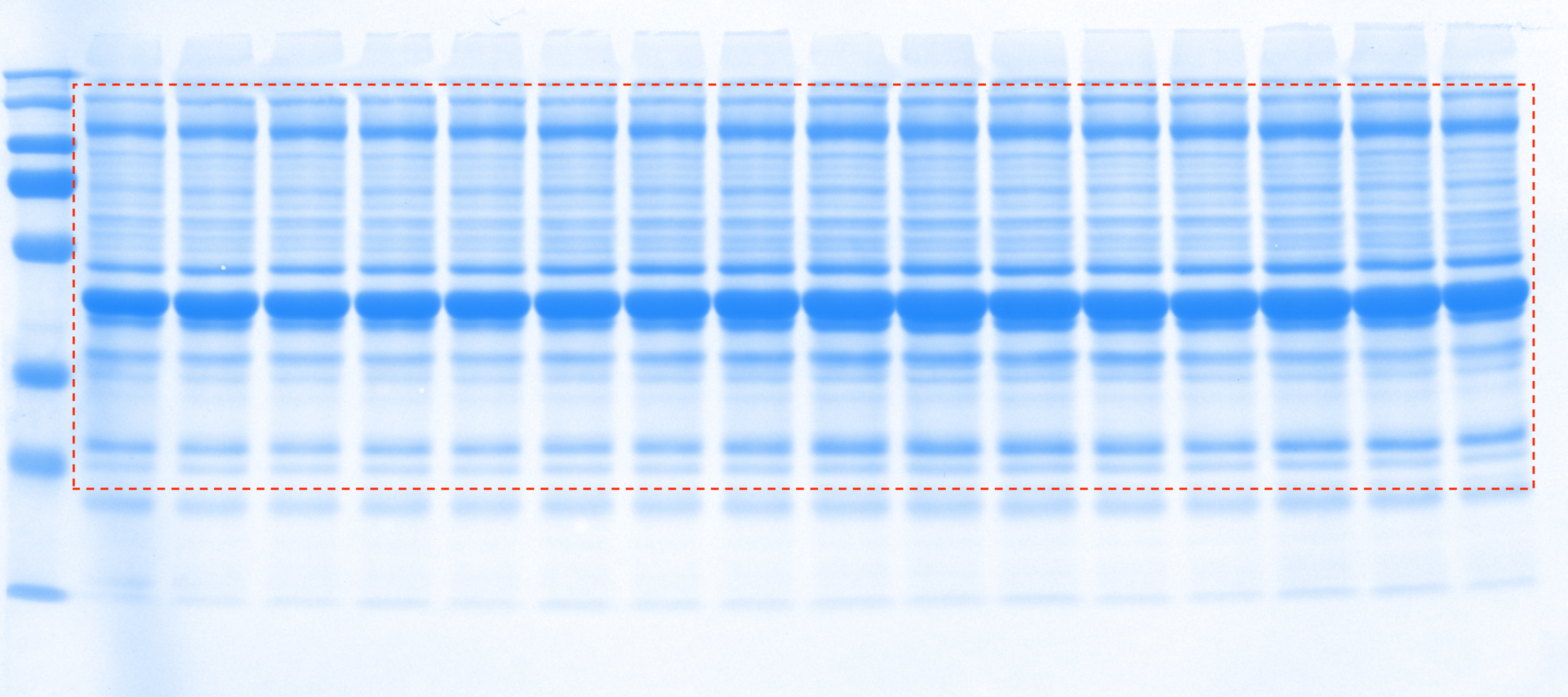

Supplement: Supplementary file 7 — Source data Fig. 3 [file 44319_2025_578_MOESM7_ESM.zip › Figure 3/3F/Coomassie stained GLUT4 blot.tif]

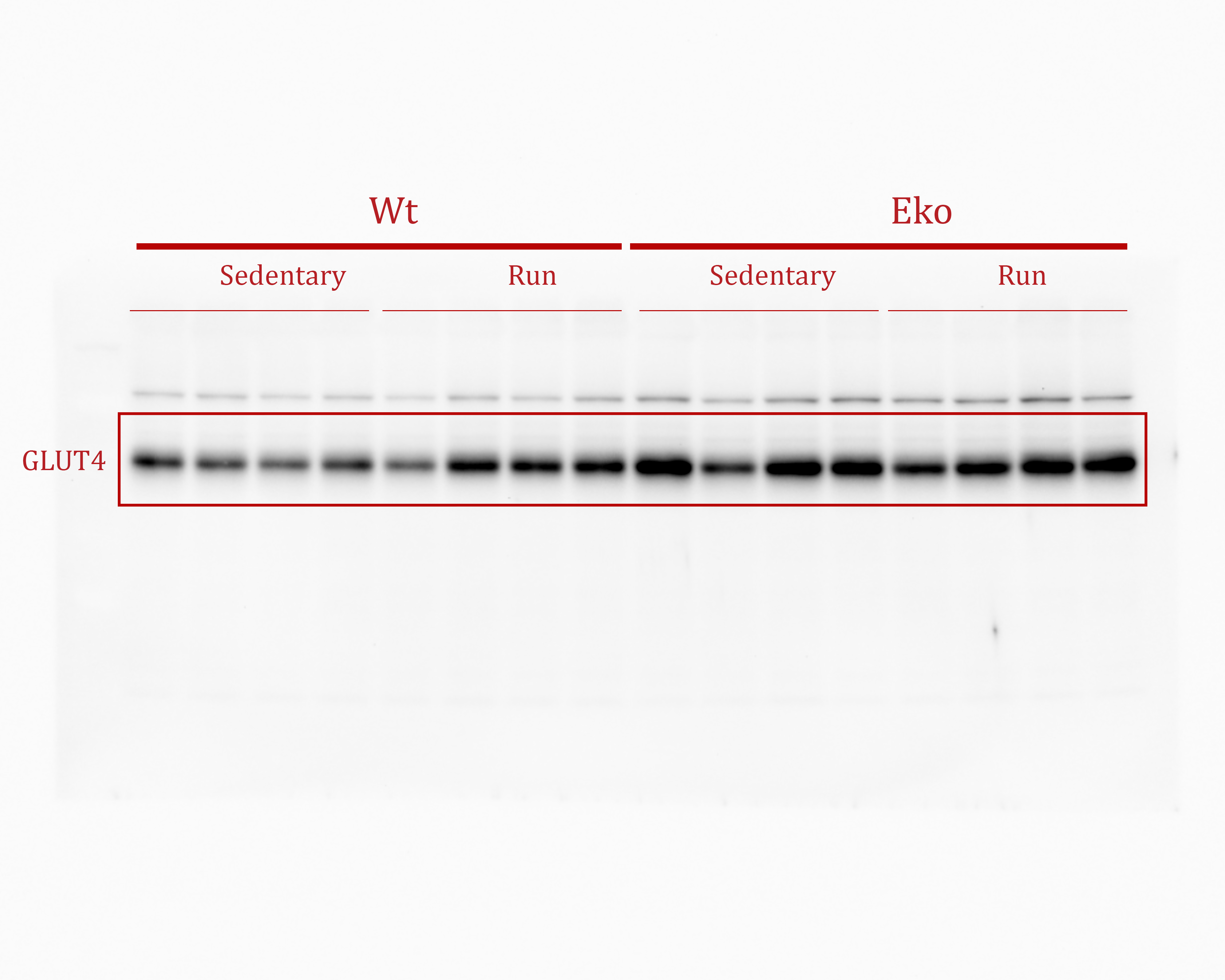

Supplement: Supplementary file 7 — Source data Fig. 3 [file 44319_2025_578_MOESM7_ESM.zip › Figure 3/3F/GLUT4 Western blot.tif]

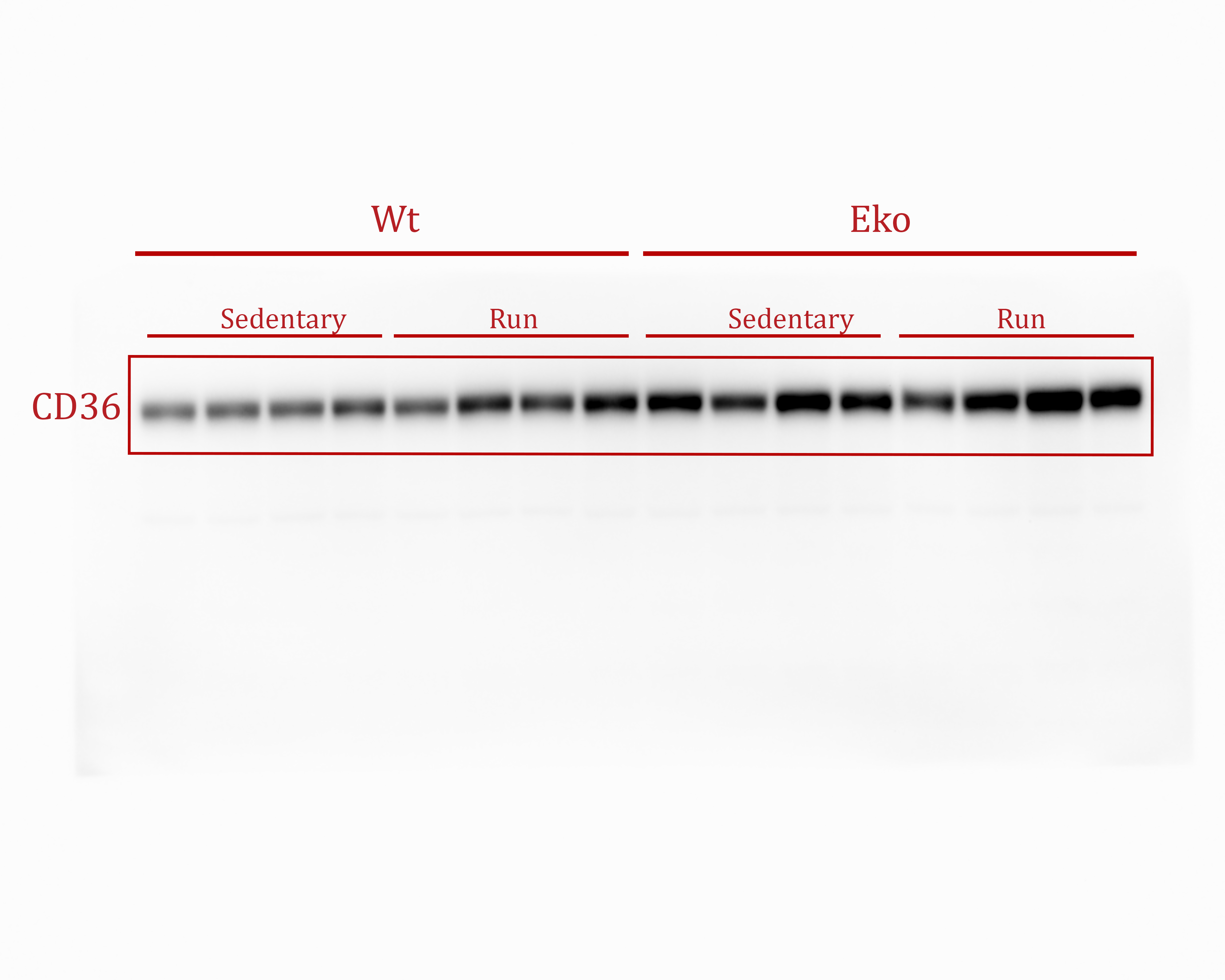

Supplement: Supplementary file 7 — Source data Fig. 3 [file 44319_2025_578_MOESM7_ESM.zip › Figure 3/3G/CD36 Western blot.tif]

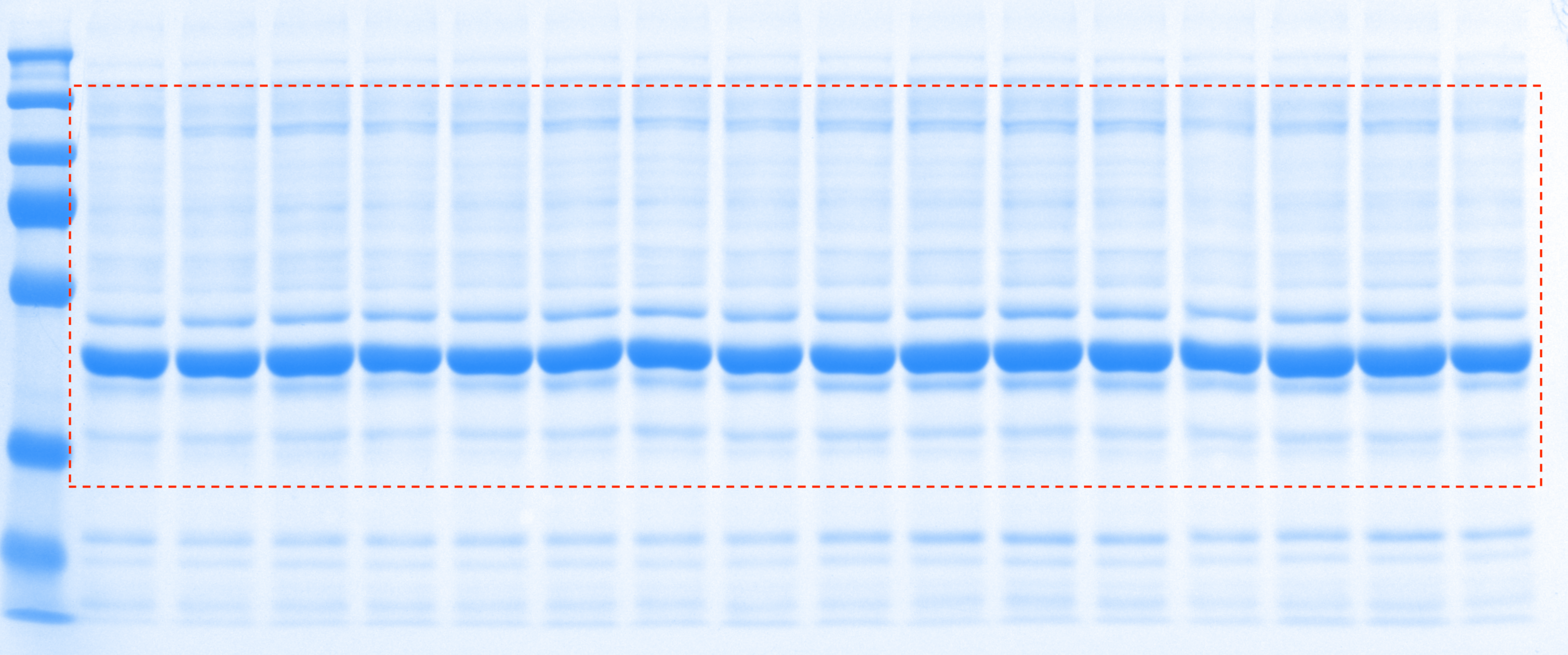

Supplement: Supplementary file 7 — Source data Fig. 3 [file 44319_2025_578_MOESM7_ESM.zip › Figure 3/3G/Coomassie stained CD36 blot.tif]

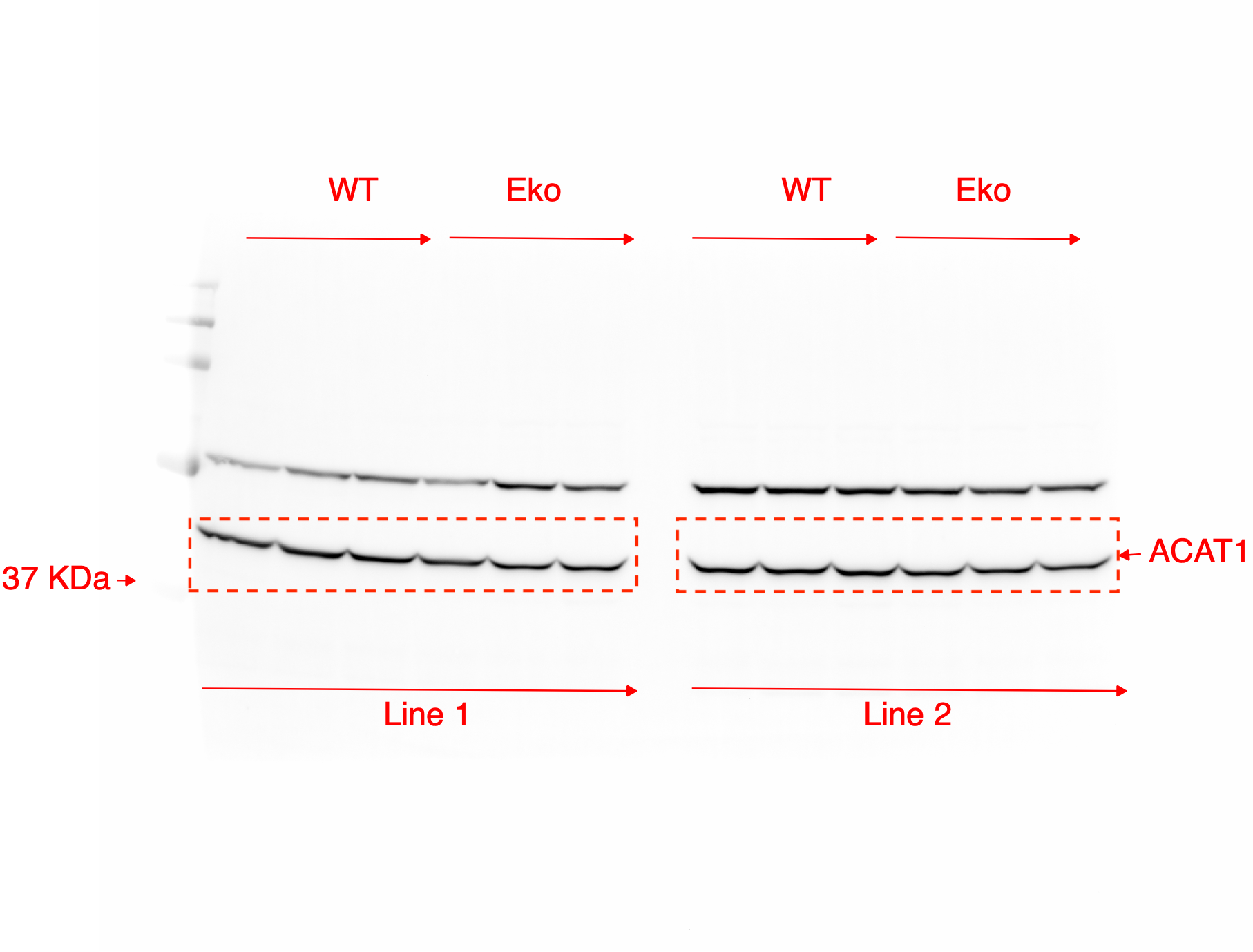

Supplement: Supplementary file 8 — Source data Fig. 4 [file 44319_2025_578_MOESM8_ESM.zip › Figure 4/4D/ACAT1 Western Blot Line 1 and 2.tif]

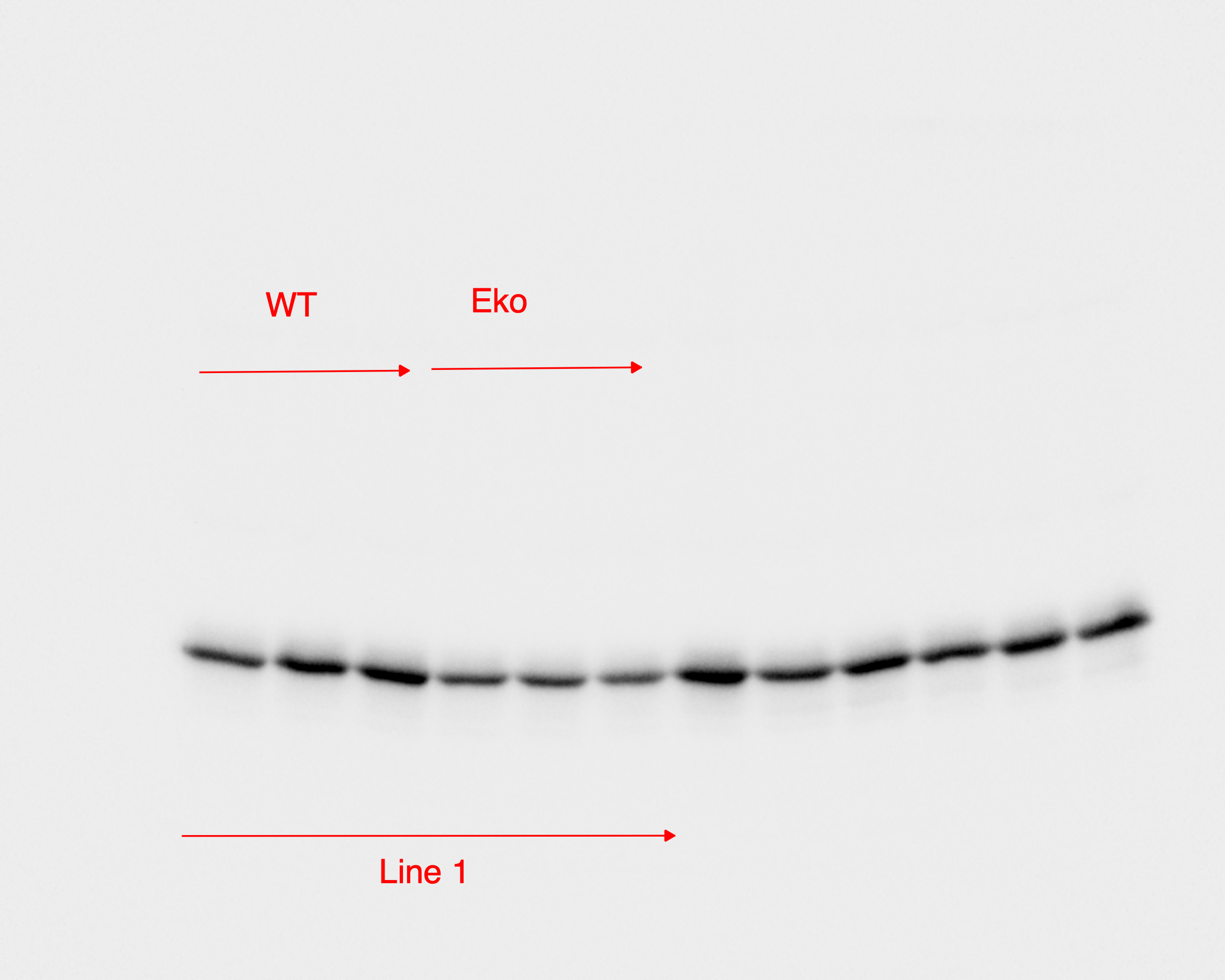

Supplement: Supplementary file 8 — Source data Fig. 4 [file 44319_2025_578_MOESM8_ESM.zip › Figure 4/4D/Bdh1_Line 1 Blot_2022-11-30 Chemiluminescence 1.000s.tif]

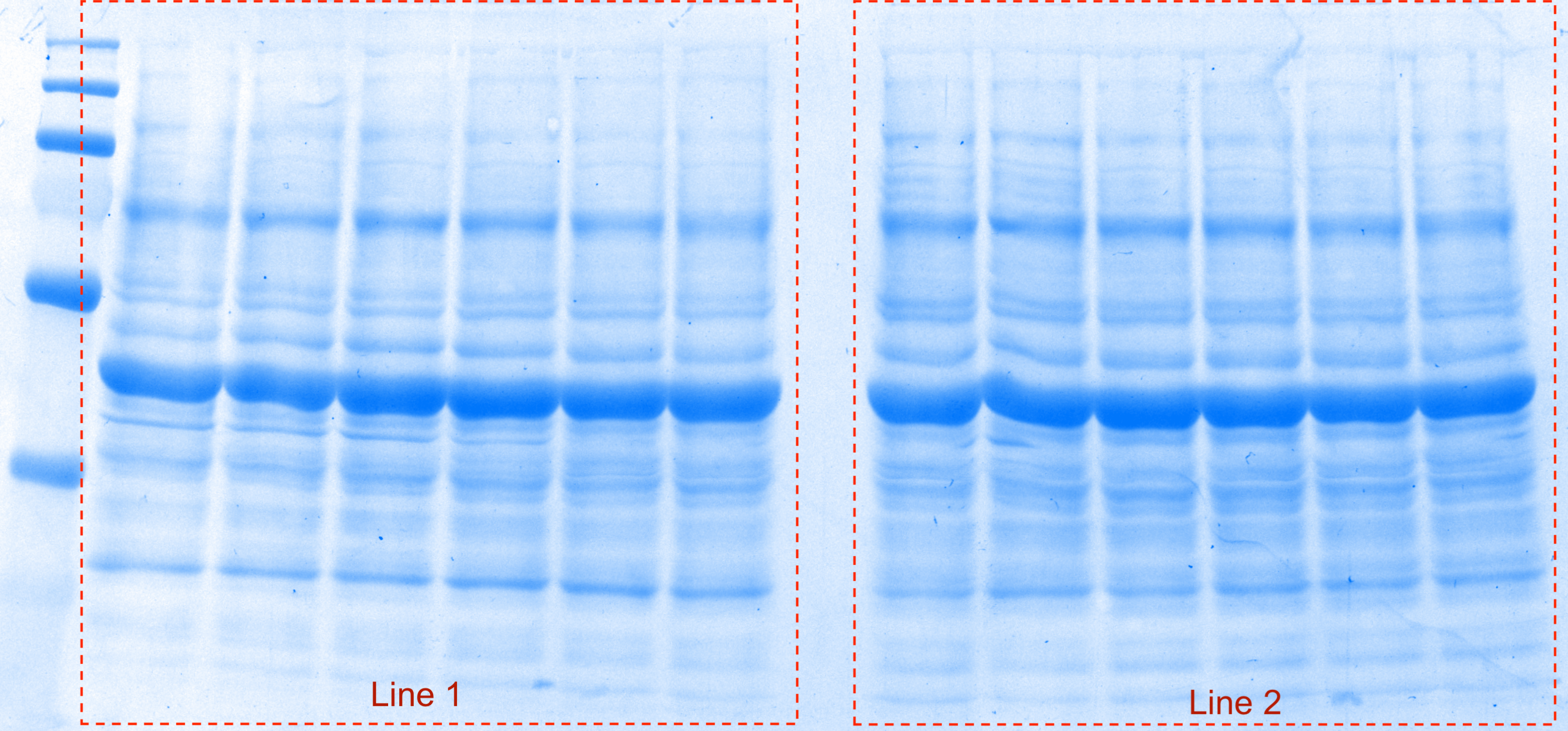

Supplement: Supplementary file 8 — Source data Fig. 4 [file 44319_2025_578_MOESM8_ESM.zip › Figure 4/4D/Oxct1 coomassie line 1 and 2.tif]

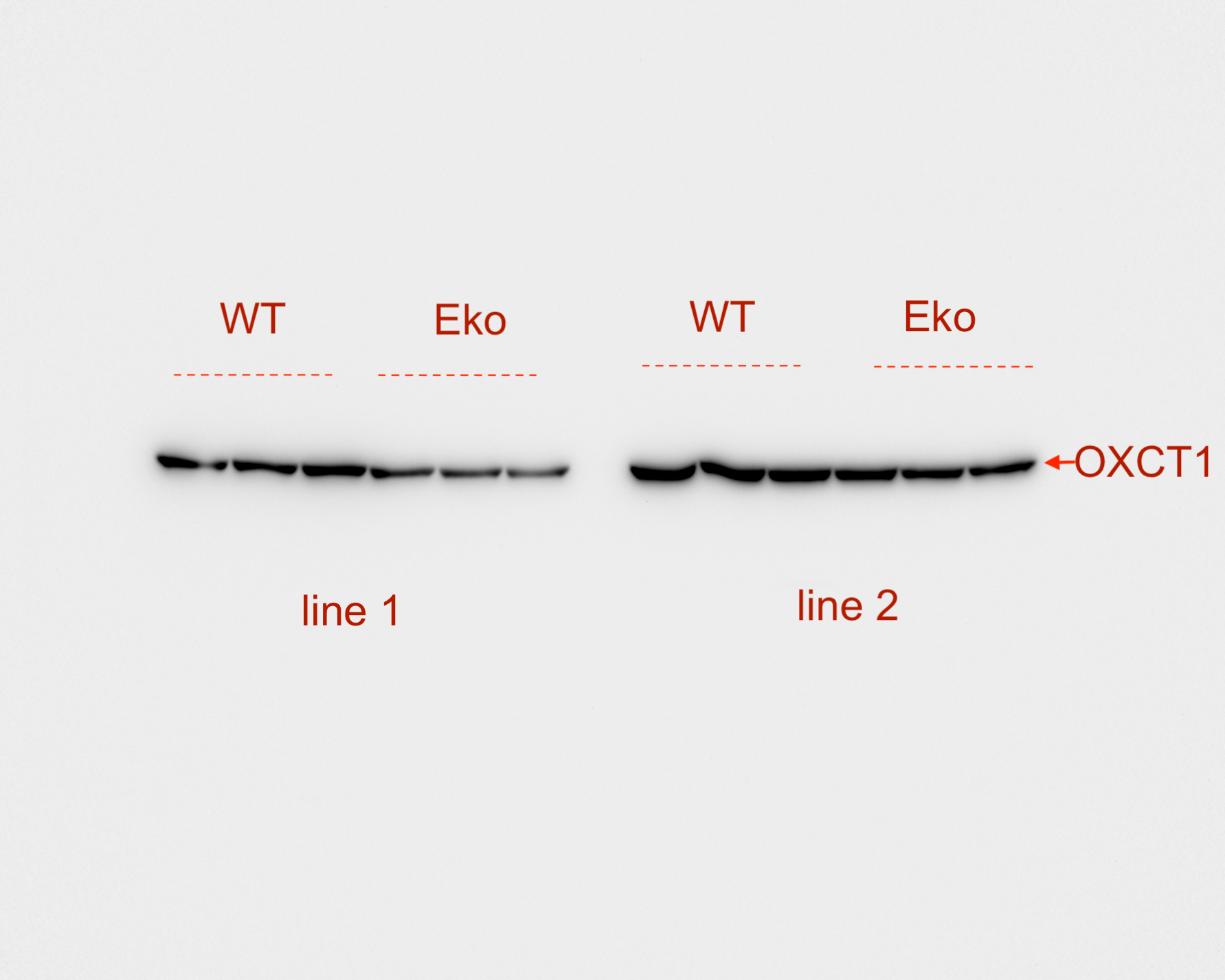

Supplement: Supplementary file 8 — Source data Fig. 4 [file 44319_2025_578_MOESM8_ESM.zip › Figure 4/4D/OXCT1 blot line 1 and 2.tif]

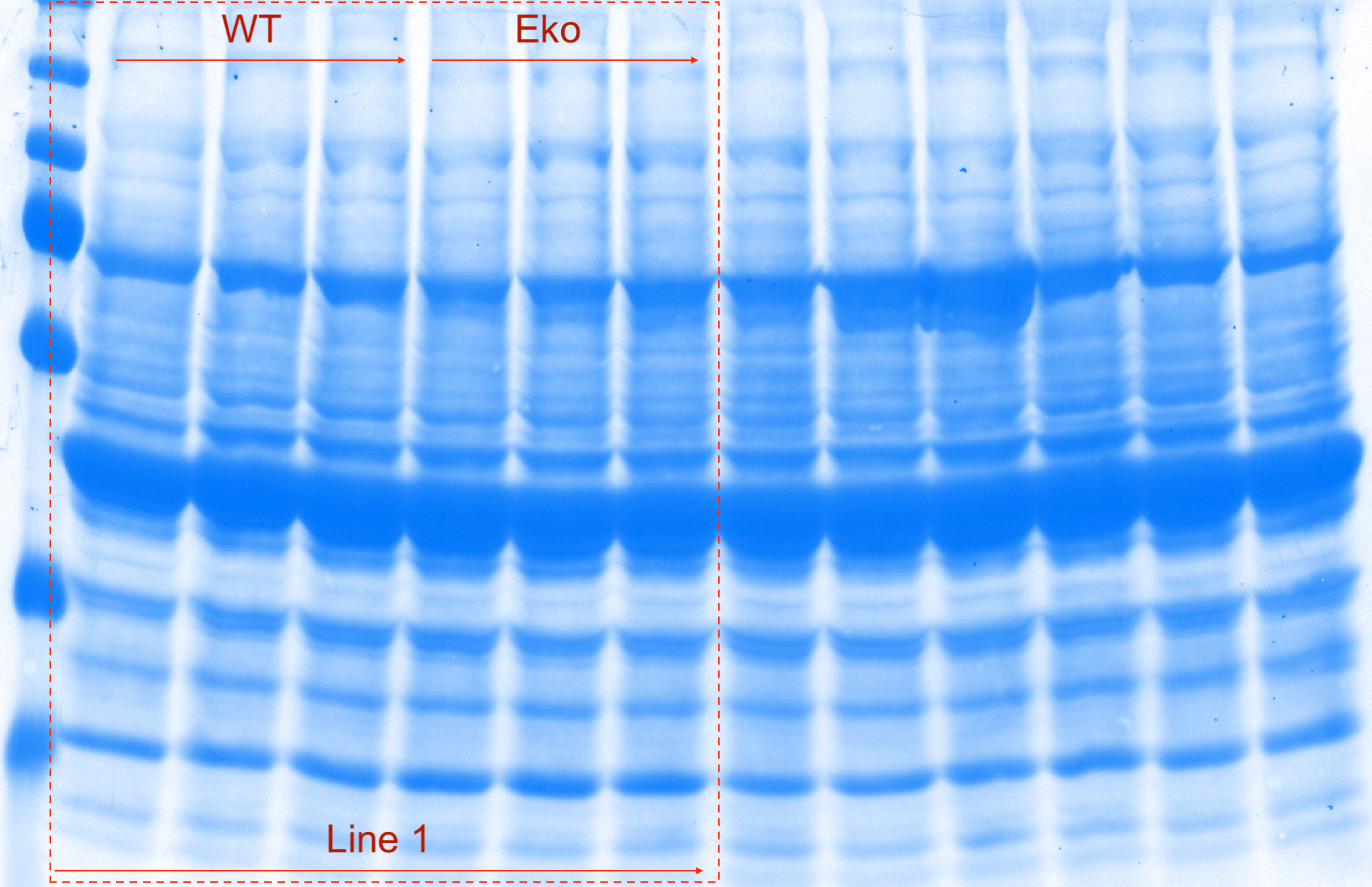

Supplement: Supplementary file 8 — Source data Fig. 4 [file 44319_2025_578_MOESM8_ESM.zip › Figure 4/4D/Bdh1 coomassie Line 1.tiff]

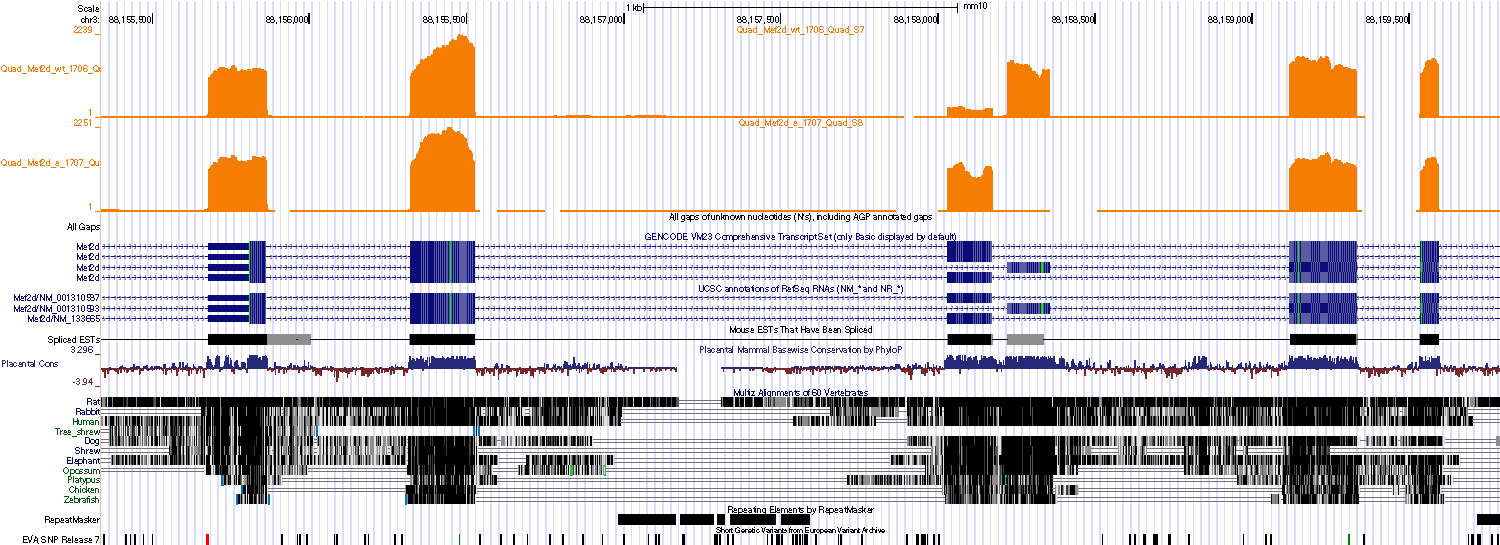

Supplement: Supplementary file 8 — Source data Fig. 4 [file 44319_2025_578_MOESM8_ESM.zip › Figure 4/4A/MEF2D tracks mapped to mm10_UCSC.tiff]

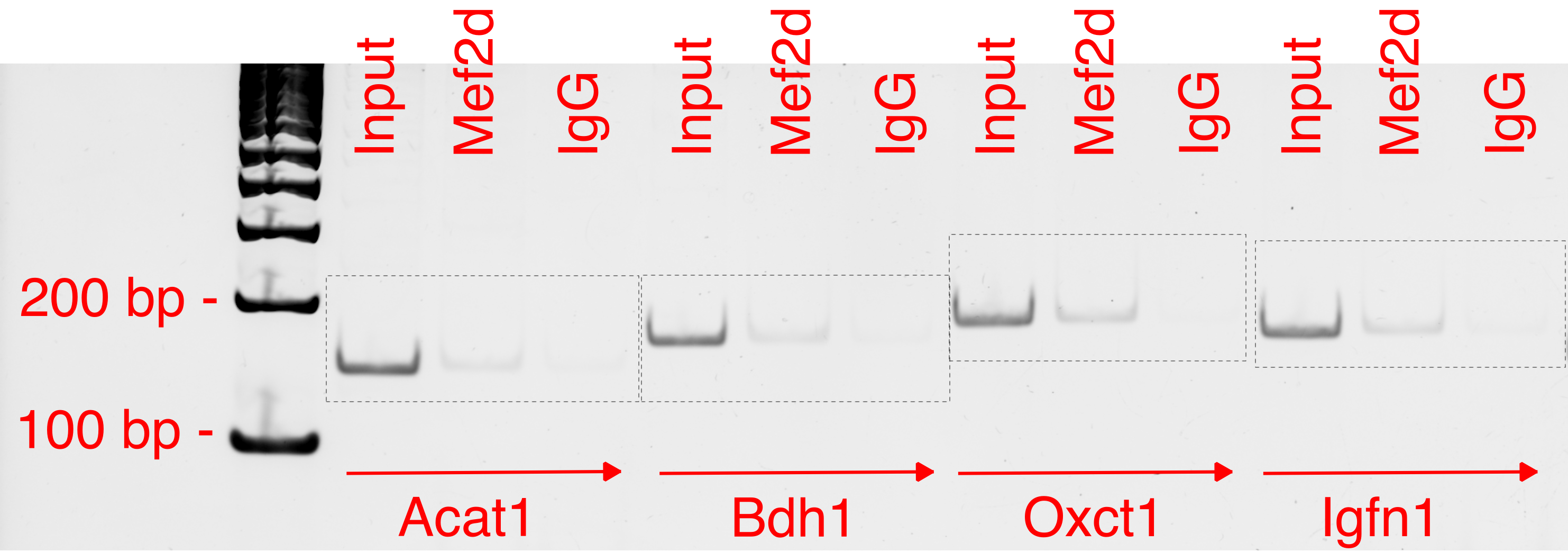

Supplement: Supplementary file 9 — Source data Fig. 5 [file 44319_2025_578_MOESM9_ESM.zip › Figure 5/5E/ChIP gel_annotated.tif]

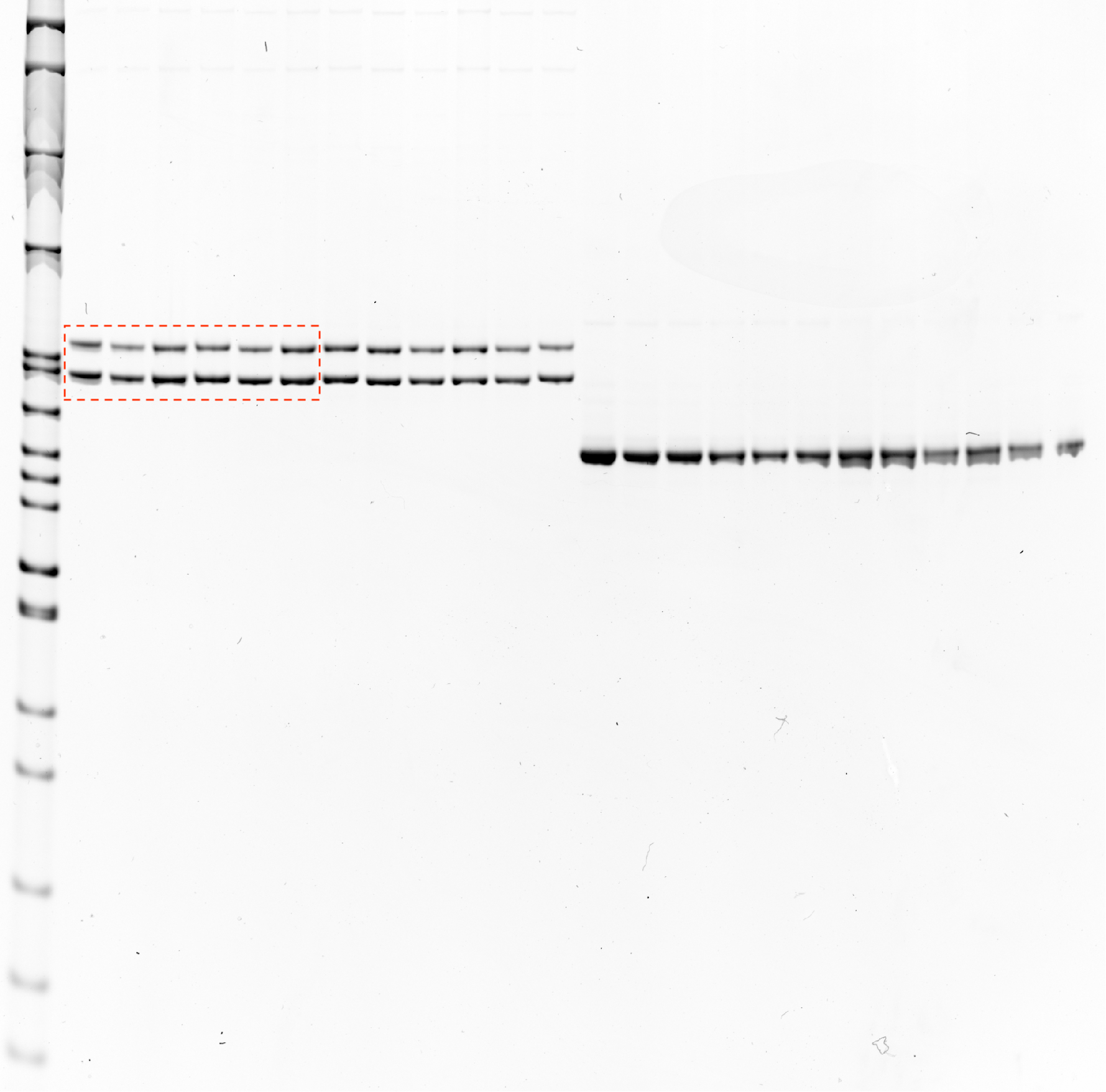

Supplement: Supplementary file 10 — Figure EV1 Source Data [file 44319_2025_578_MOESM10_ESM.zip › Figure EV1/EV1C/gas mef2a alpha.tif]

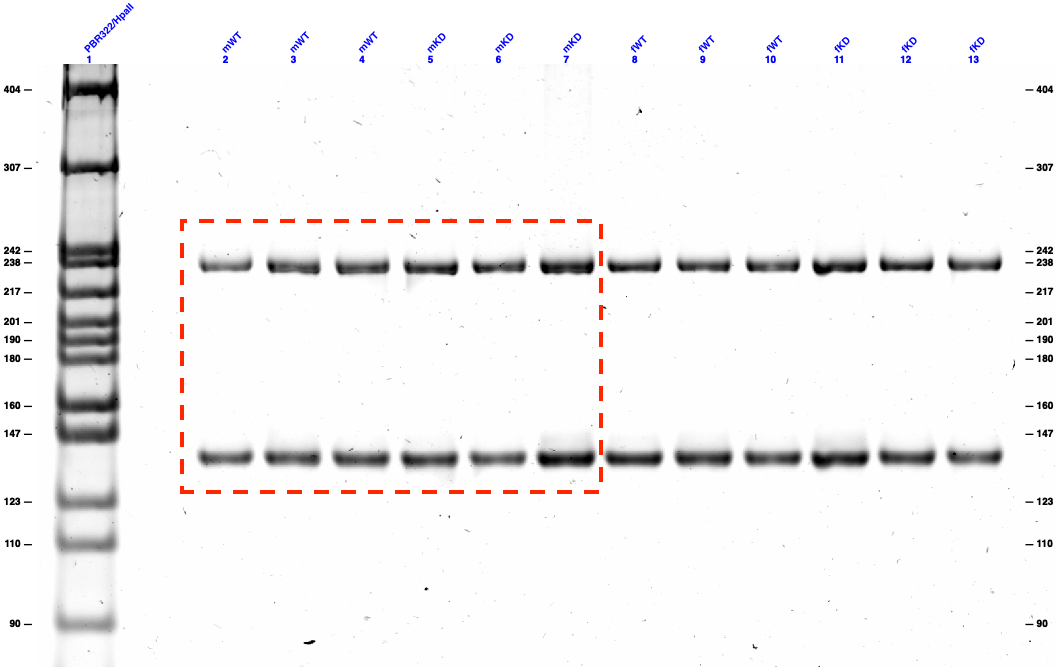

Supplement: Supplementary file 10 — Figure EV1 Source Data [file 44319_2025_578_MOESM10_ESM.zip › Figure EV1/EV1C/gas mef2c gamma.tif]

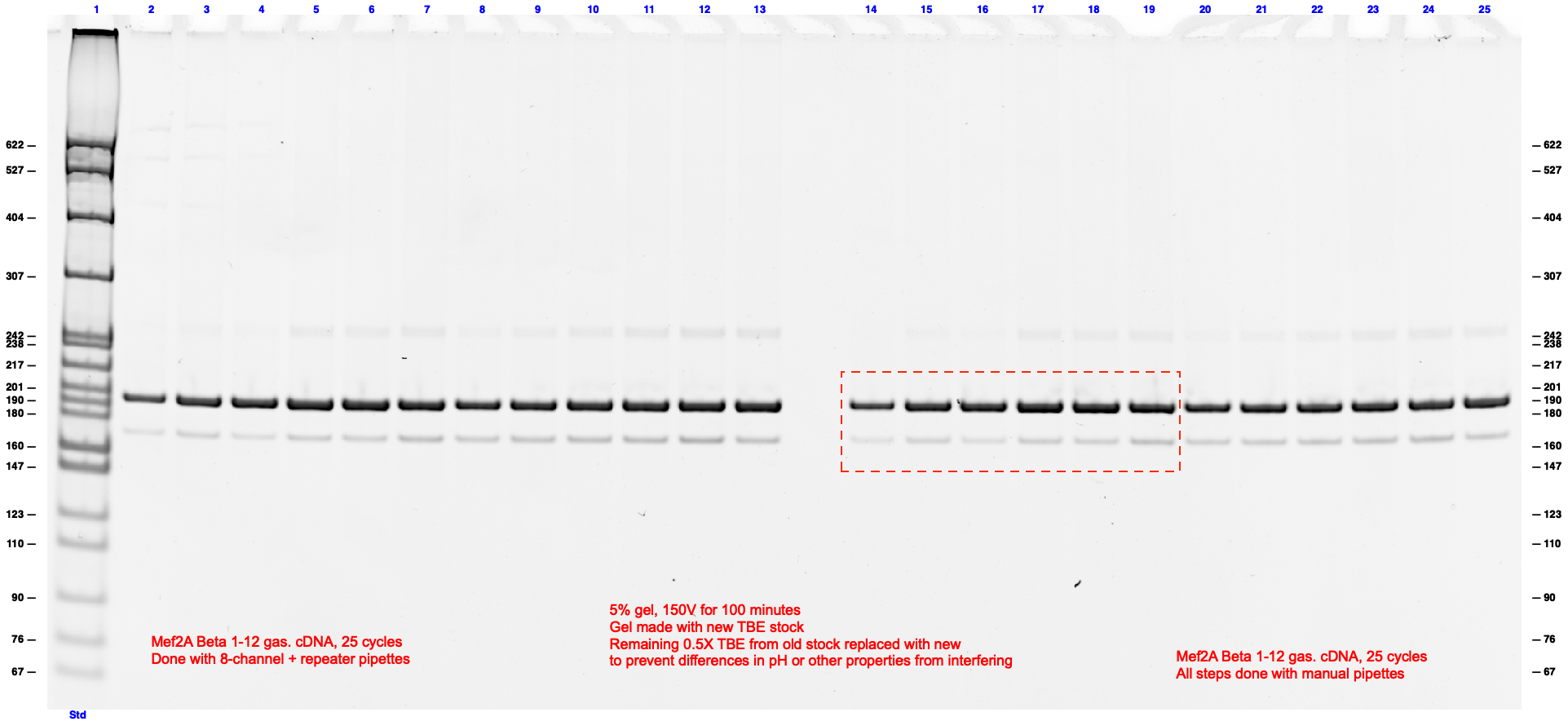

Supplement: Supplementary file 10 — Figure EV1 Source Data [file 44319_2025_578_MOESM10_ESM.zip › Figure EV1/EV1C/gas mef2a beta.tif]

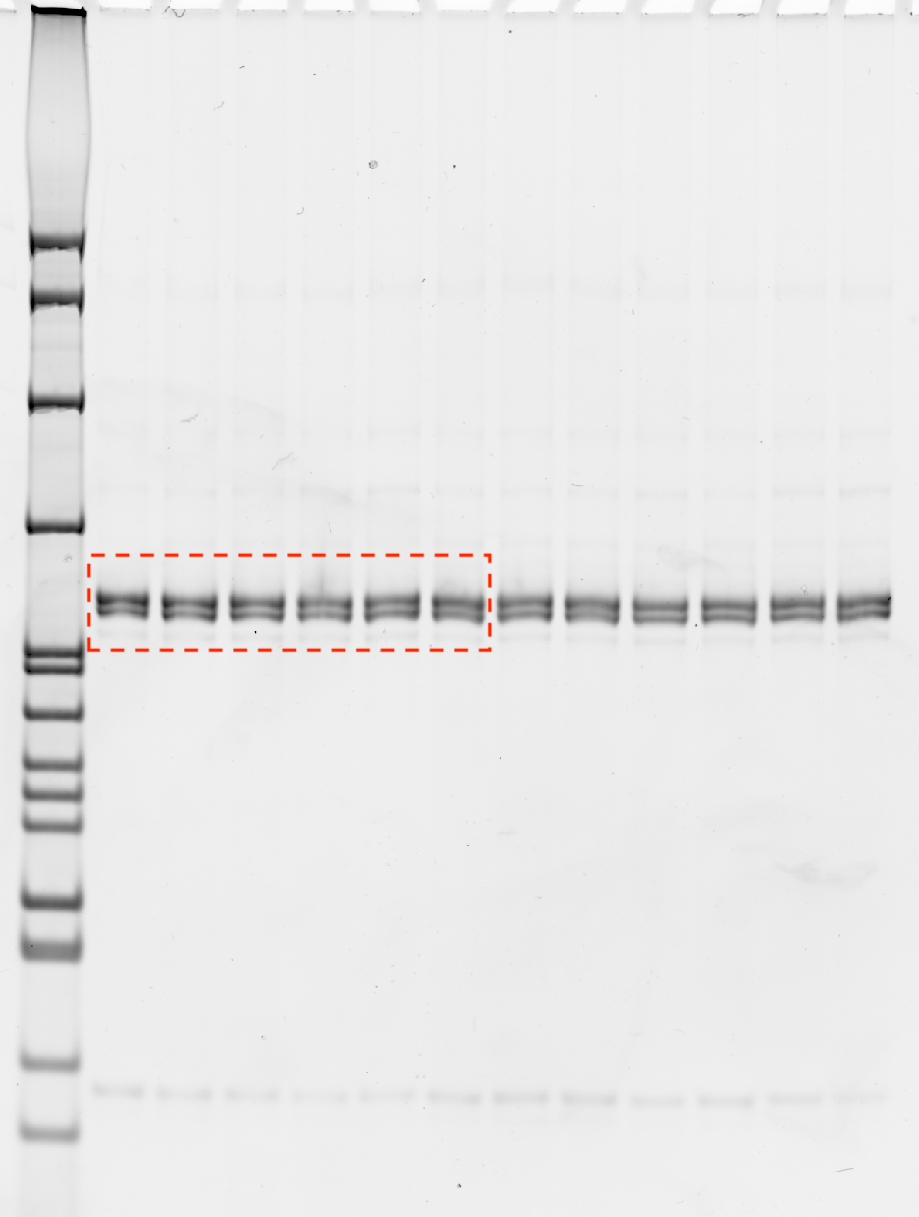

Supplement: Supplementary file 10 — Figure EV1 Source Data [file 44319_2025_578_MOESM10_ESM.zip › Figure EV1/EV1C/gas mef2c alpha.tif]

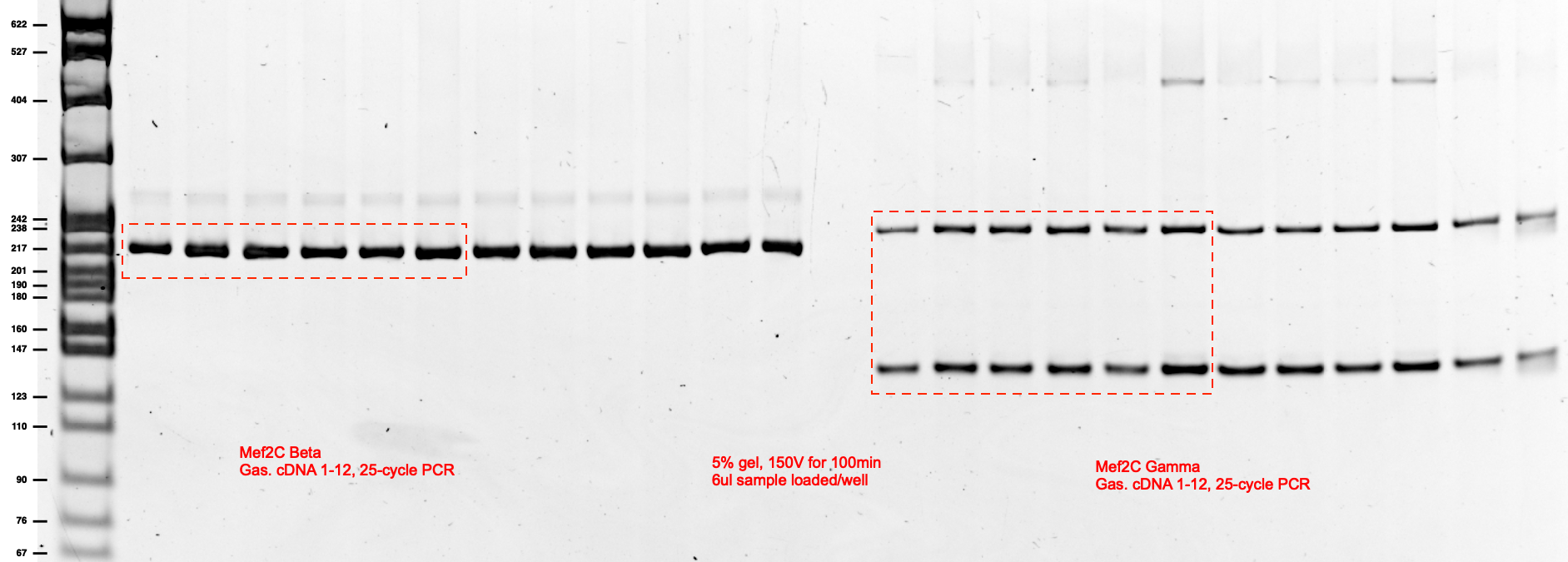

Supplement: Supplementary file 10 — Figure EV1 Source Data [file 44319_2025_578_MOESM10_ESM.zip › Figure EV1/EV1C/gas mef2c beta.tif]

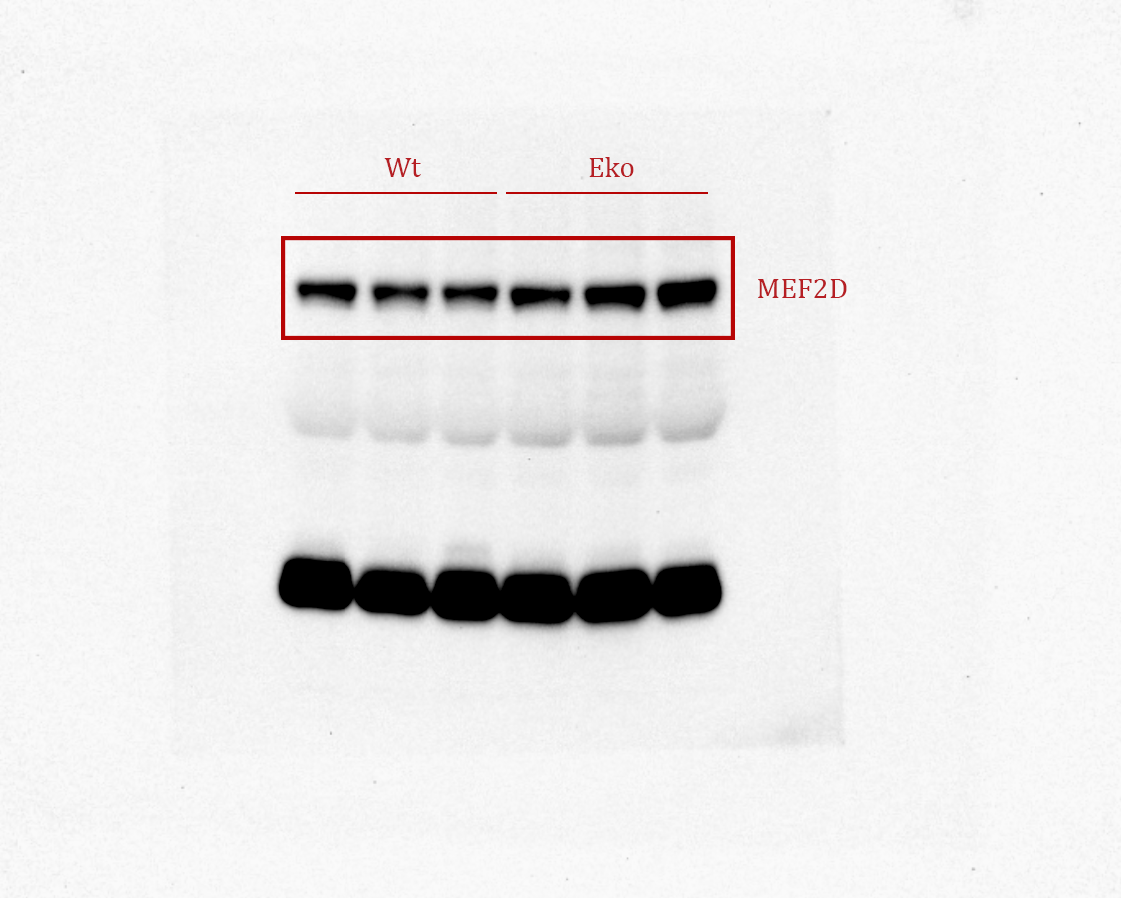

Supplement: Supplementary file 10 — Figure EV1 Source Data [file 44319_2025_578_MOESM10_ESM.zip › Figure EV1/EV1D/MEF2D Western blot-Tibialis Anterior.tif]

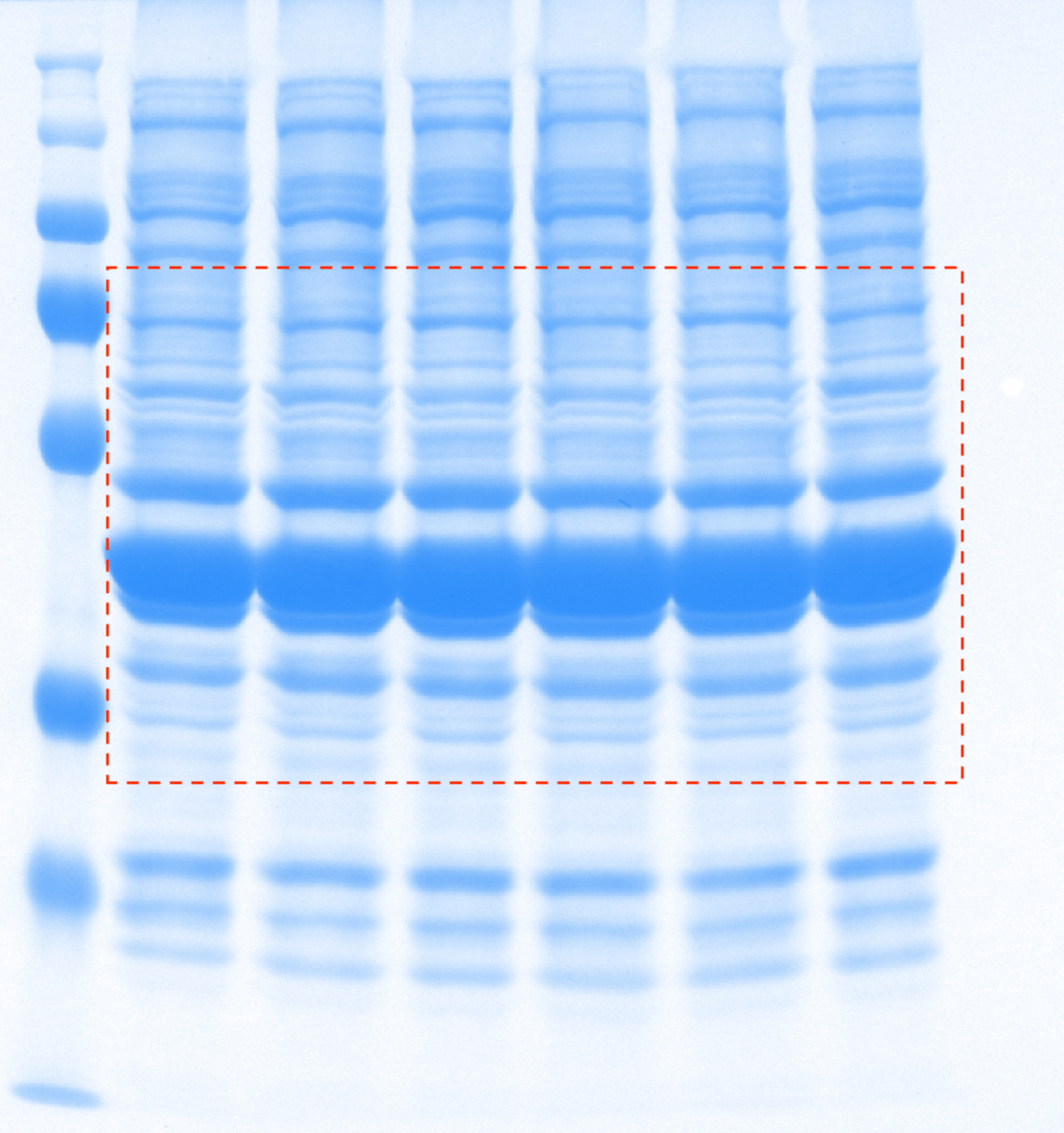

Supplement: Supplementary file 10 — Figure EV1 Source Data [file 44319_2025_578_MOESM10_ESM.zip › Figure EV1/EV1D/Coomassie stained MEF2D-Western Blot-Tibialis Anterior.tif]

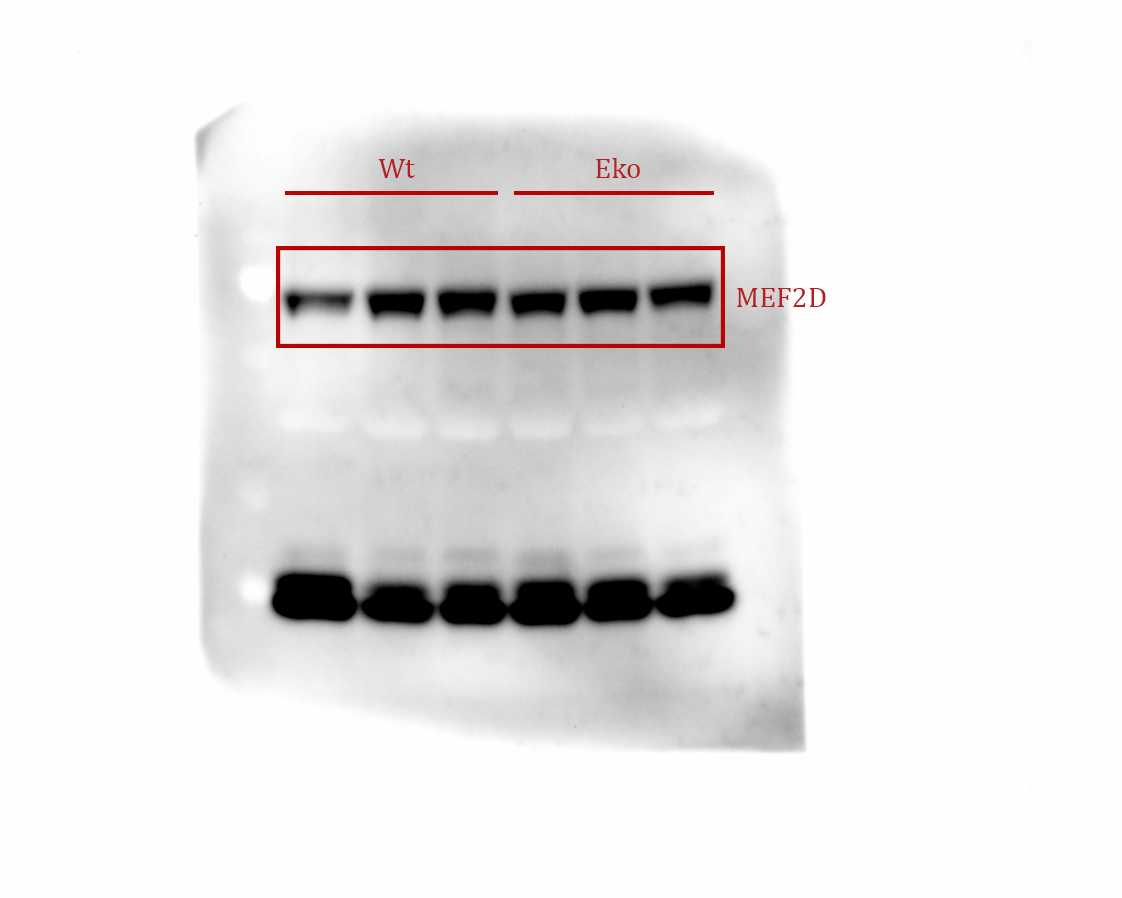

Supplement: Supplementary file 10 — Figure EV1 Source Data [file 44319_2025_578_MOESM10_ESM.zip › Figure EV1/EV1D/MEF2D-Western Blot-Soleus.tif]

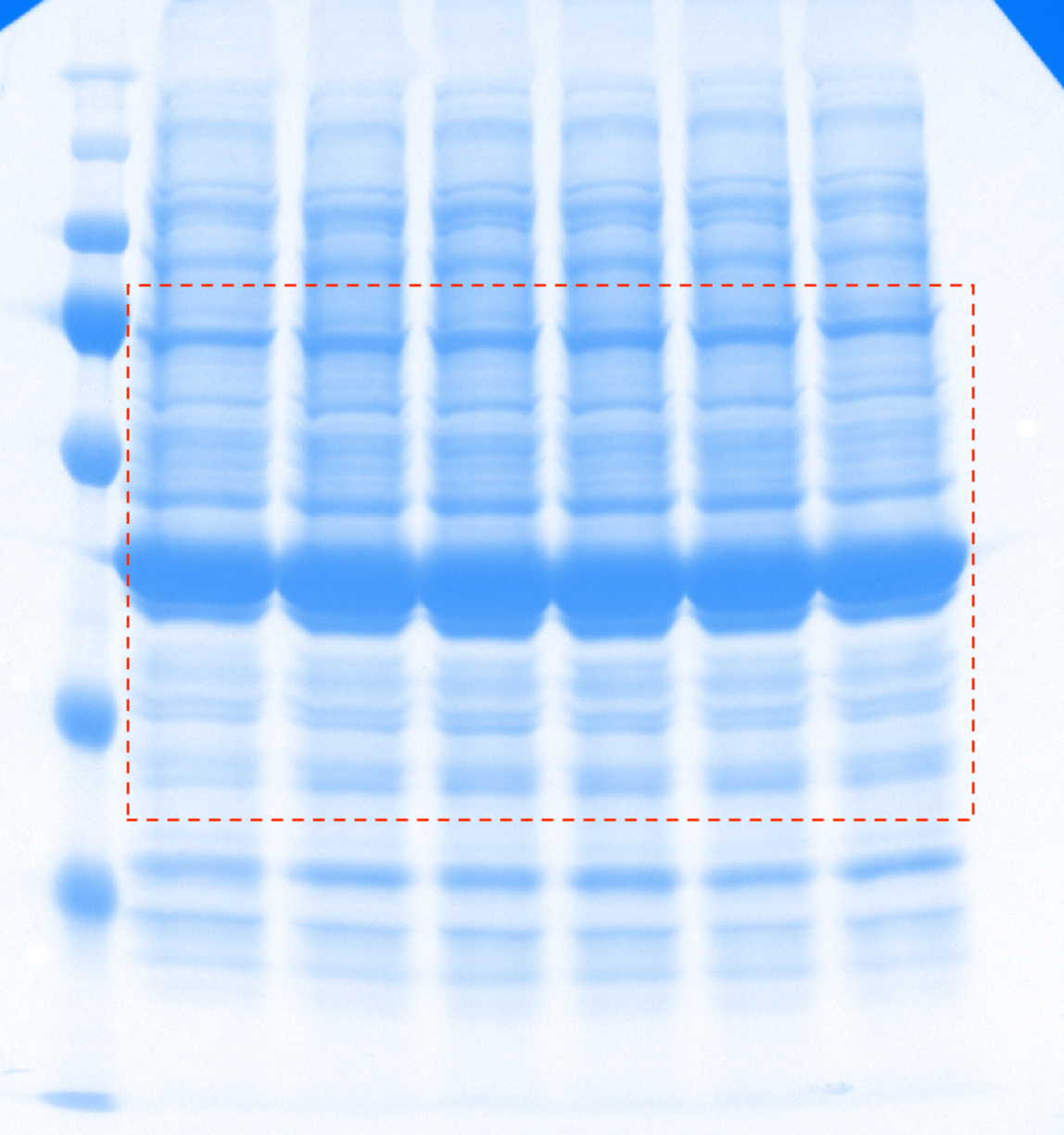

Supplement: Supplementary file 10 — Figure EV1 Source Data [file 44319_2025_578_MOESM10_ESM.zip › Figure EV1/EV1D/Coomassie stained MEF2D-Western Blot-Soleus.tif]

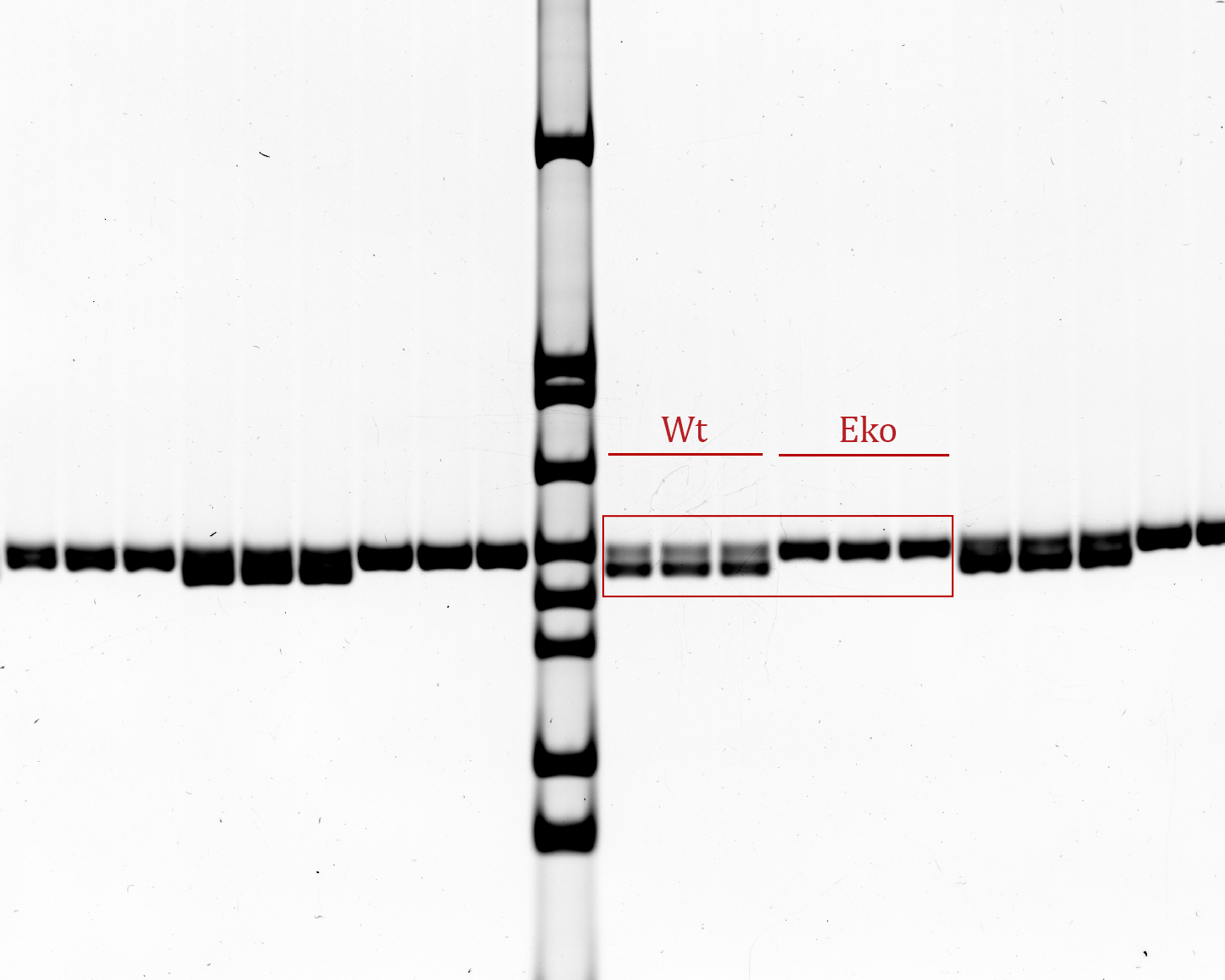

Supplement: Supplementary file 10 — Figure EV1 Source Data [file 44319_2025_578_MOESM10_ESM.zip › Figure EV1/EV1B/Mef2d alpha-splicing gel_Soleus.tif]

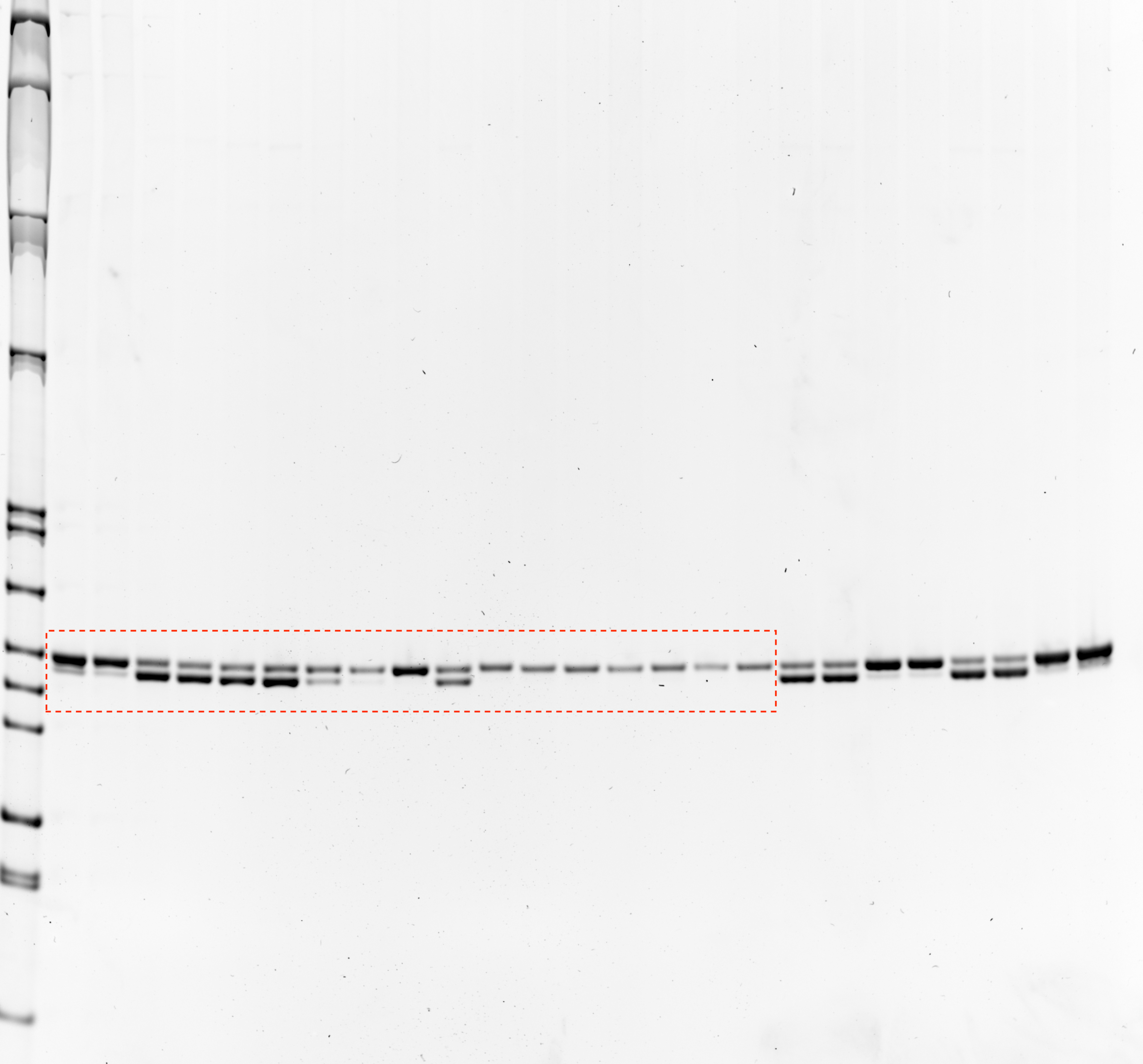

Supplement: Supplementary file 10 — Figure EV1 Source Data [file 44319_2025_578_MOESM10_ESM.zip › Figure EV1/EV1A/Fig EV1a.tif]

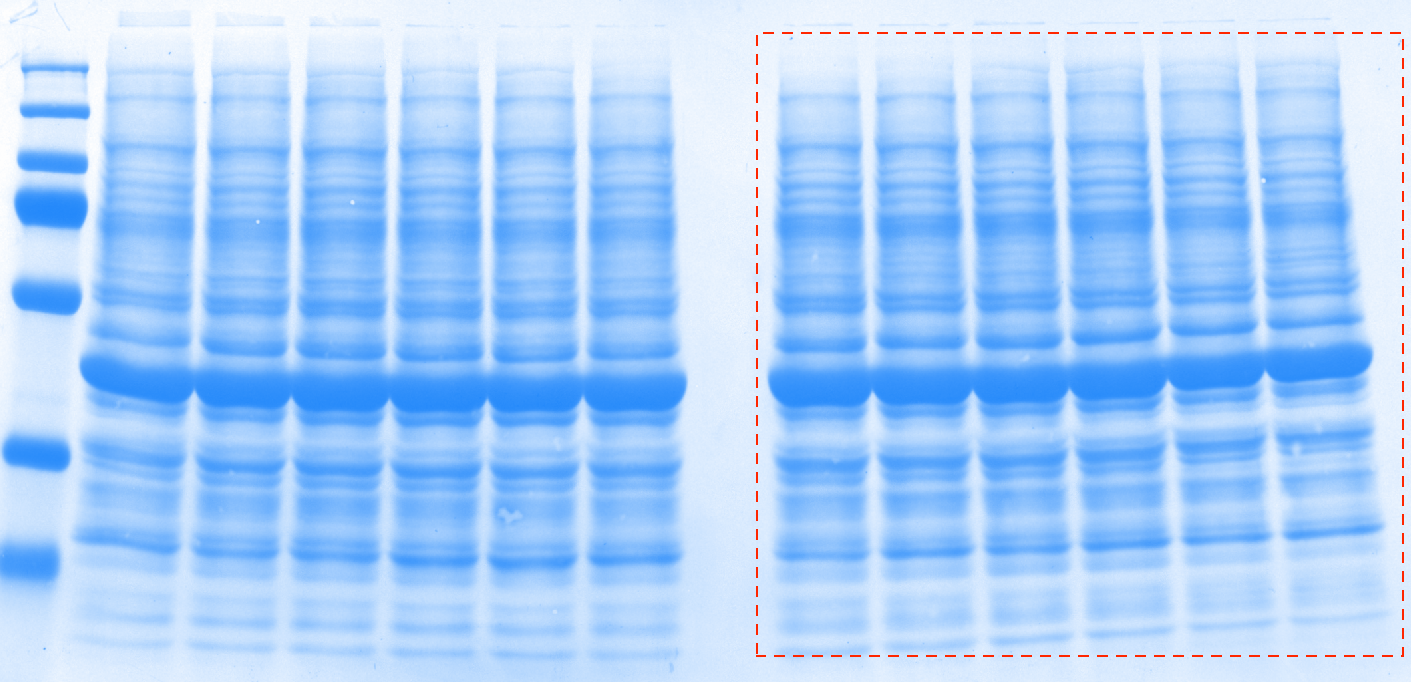

Supplement: Supplementary file 13 — Figure EV4 Source Data [file 44319_2025_578_MOESM13_ESM.zip › Figure EV4/EV4 E/Bdh1 Line2 coommassie.tif]

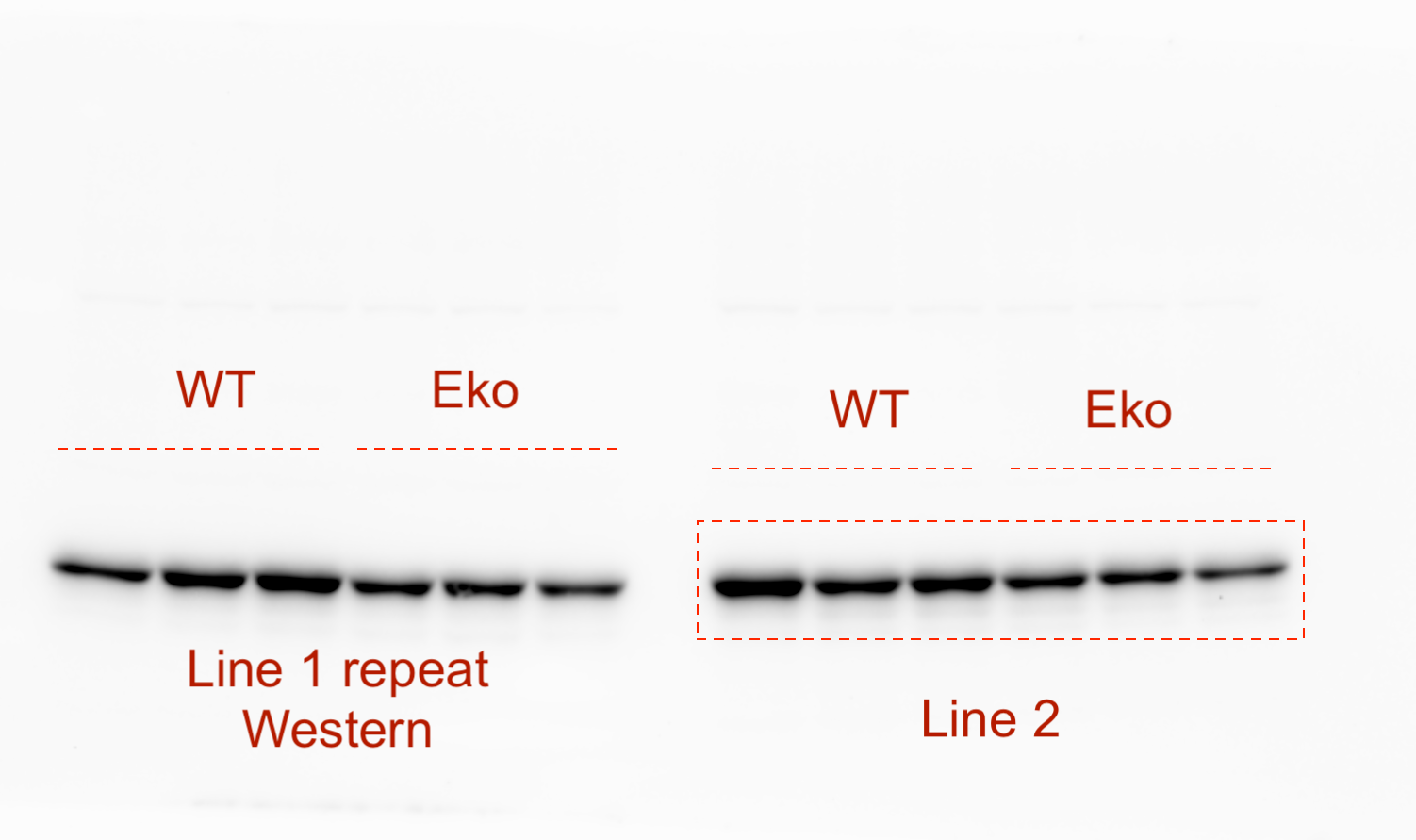

Supplement: Supplementary file 13 — Figure EV4 Source Data [file 44319_2025_578_MOESM13_ESM.zip › Figure EV4/EV4 E/Bdh1 line 2.tif]

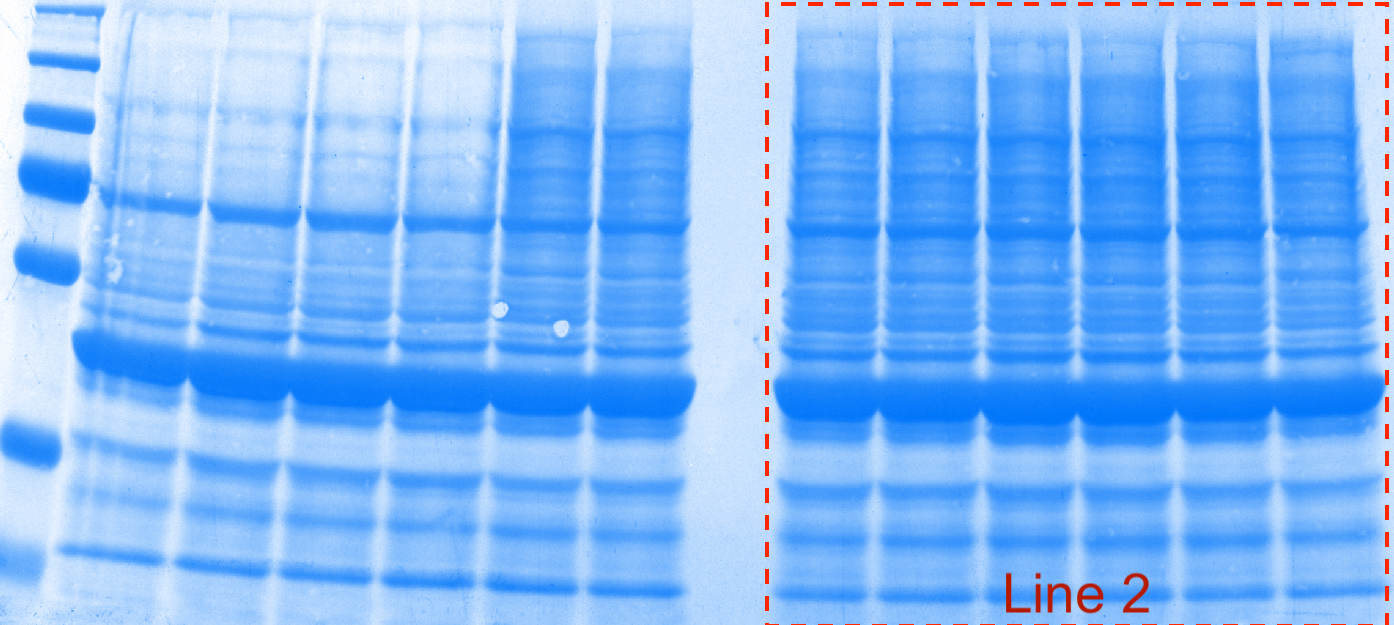

Supplement: Supplementary file 13 — Figure EV4 Source Data [file 44319_2025_578_MOESM13_ESM.zip › Figure EV4/EV4 E/ACAT1 coomassie Line 1 and 2.tif]
