# Supplementary material for: The muscle specific MEF2Dα2 isoform promotes muscle ketolysis and running capacity in mice
Source: EMBO Rep. 2025 Sep 16;26(21):5216–38. doi: 10.1038/s44319-025-00578-3 (PMC12592725; doi:10.1038/s44319-025-00578-3)
Supplement: Supplementary file 15 — Expanded View Figures [file 44319_2025_578_MOESM15_ESM.pdf]

Expanded View Figures

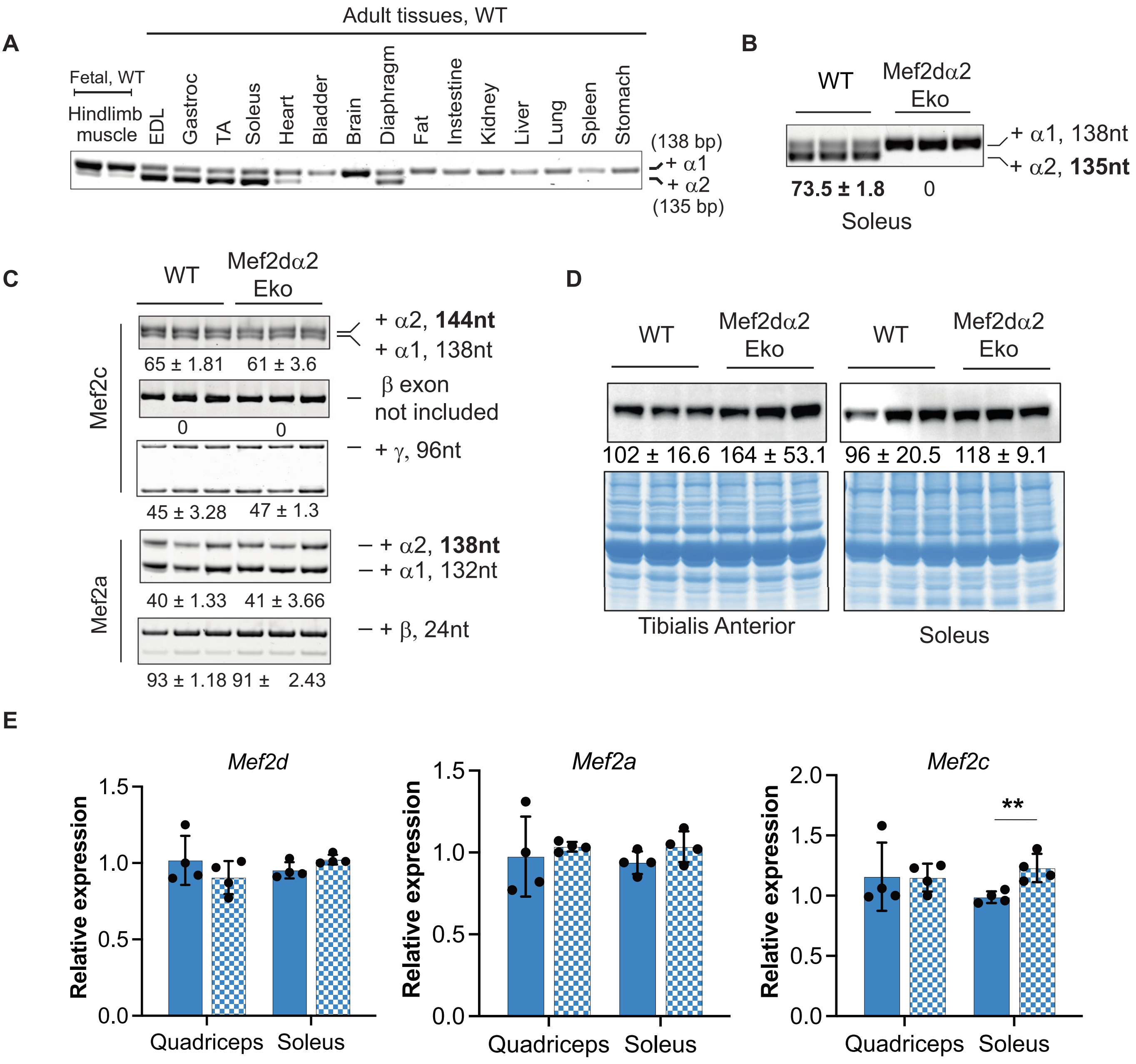

Figure EV1. Deletion of Mef2dα2 exon does not affect the expression or splicing of other MEF2 genes.

(**A**) RT-PCR analysis of the mutually exclusive α-exons in *Mef2d* transcript using total RNA from the indicated fetal hind limb muscle and tissues from adult wild-type mice. (**B**) RT-PCR analysis *of Mef2d* α exons using soleus RNA from line 2. The numbers indicate PSI; data are mean ± SD; $n = 3$. The numbers indicate the percent spliced in (PSI). Bolded numbers are significant by Student *T* test. (**C**) RT-PCR analysis of the alternative exons in *Mef2c* and *Mef2a* transcripts using total RNA from gastrocnemius muscle from line 1. Data are mean ± SD; $n = 3$. PSI for exon is indicated and was not significantly different between the genotypes by Student's *t* test. (**D**) Western blot showing MEF2D protein levels in TA (upper left) and Soleus muscles (upper right) from line 2. The bottom panels show Coomassie stained blots, from the upper panels showing total protein loaded. The numbers are mean ± SD; $n = 3$. Student's *t* test found no differences in genotypes. (**E**) RT-qPCR showing relative mRNA levels *Mef2d*, *Mef2a*, and *Mef2c* relative to *Rpl30* using total RNA in the indicated muscle groups from line 2 mice. Data are mean ± SEM; $n = 4$. $^{**}P = 0.0088$ (multiple Student's *t* test, unpaired). Source data are available online for this figure.

© The Author(s)

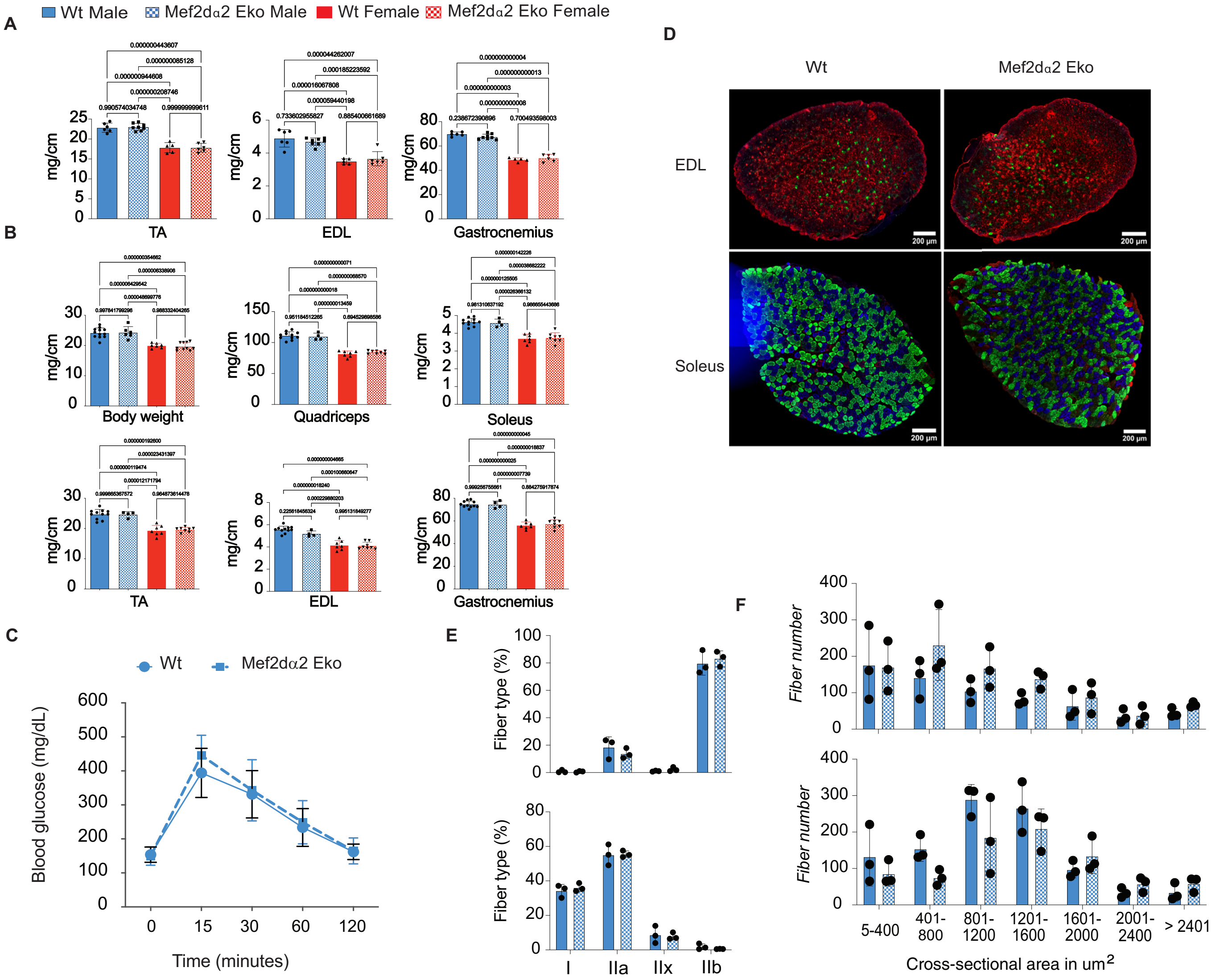

Figure EV2. No observable phenotypes in Mef2dα2 Eko mice.

(**A**) Isolated muscle weights of the indicated muscle groups from line 1 mice normalized to tibia length. Data are mean ± SD; $n \geq 5$. ****$P < 0.0001$ by one-way ANOVA. (**B**) Body and indicated muscle weights of 7-month-old mice normalized to tibia length from line 2. Data are mean ± SD; $n \geq 4$. ****$P < 0.0001$, ***$P < 0.001$ by one-way ANOVA. (**C**) GTT in line 29-weeks-old mice from line 2. Data are mean ± SD, $n = 9$. (**D**) Representative images showing cross-sections of EDL (top) and Soleus (bottom) muscles from line 2 mice immunostained for MHC-isoforms; MYH7 (Type I, blue), MYH2 (Type IIa, green), MYH4 (Type IIb, red), and MYH1 (Type IId/x, unstained). (**E**) Quantification of fiber-type proportion in EDL (top) and soleus (Bottom). Data are mean ± SEM; $n = 3$. (**F**) Distribution of cross-sectional area of myofibers in EDL (top) and Soleus (bottom) muscles from line 2 mice. Data are mean ± SEM; $n = 3$. Multiple *t* tests were performed, and no significant genotype differences were found in (**C, E, F**). Source data are available online for this figure.

 © The Author(s)

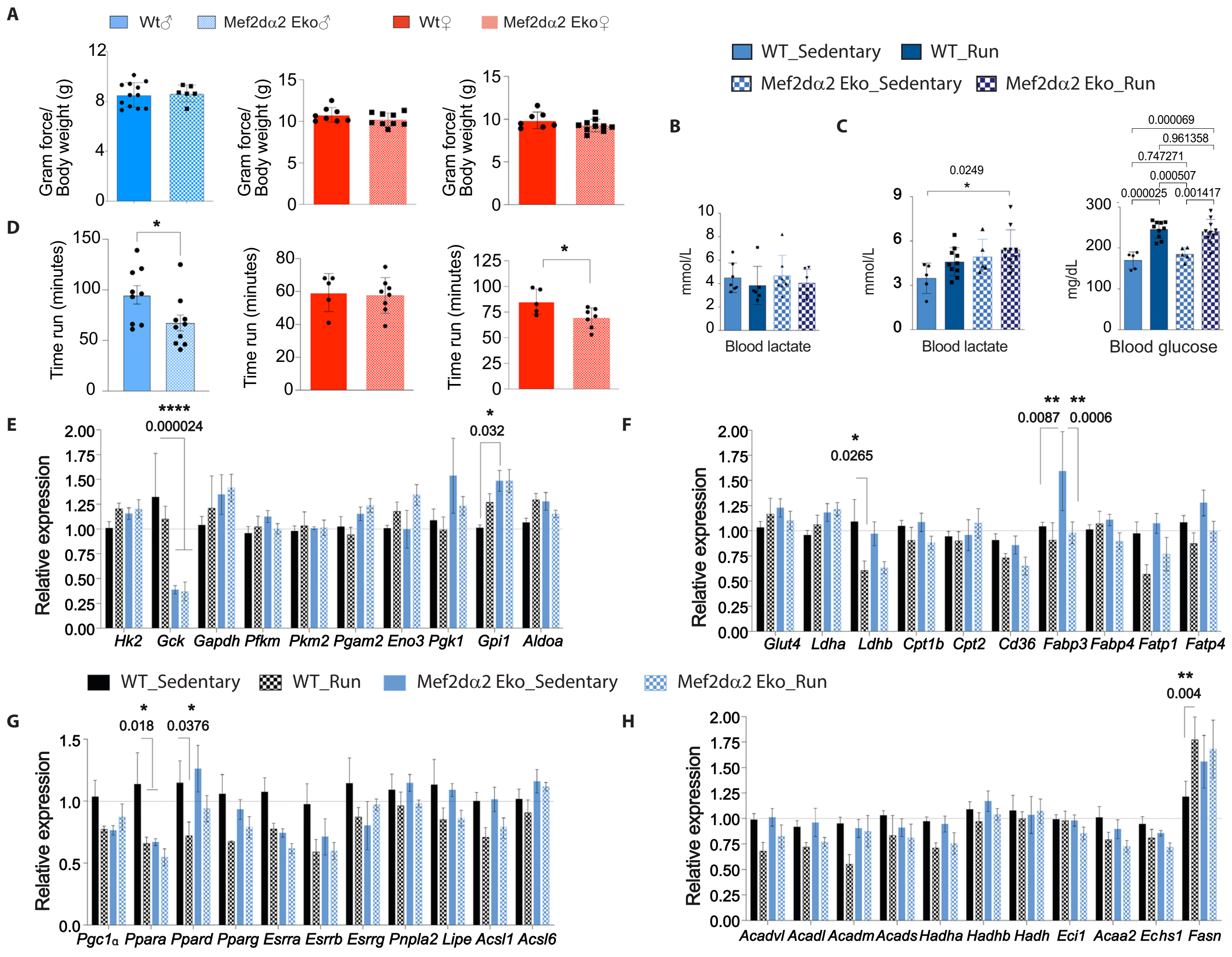

Figure EV3. Mef2dα2 Eko mice show moderate changes in muscle expression of genes involved in glucose and fatty acid metabolism.

(**A**) All limb grip strength of indicated male mice from line 2 when normalized to body weight at 9–10-weeks age (left panel, $n \geq 6$). All limb grip strength, normalized to body weight, of indicated age-matched female mice from line 1 (middle panel, $n \geq 8$) and line 2 (right panel, $n \geq 7$). Data are mean ± SD. Multiple *t* test found no differences between the genotypes. (**B**) Blood lactate levels in indicated sedentary mice and mice run at constant moderate speed till near exhaustion from line 1. Data are mean ± SD; $n \geq 5$. No genotype differences found by one-way ANOVA. (**C**) Blood lactate (left) and glucose (right) levels in indicated mice and mice run using increasing speed or high-intensity protocol. Data are mean ± SD; $n \geq 5$. The exact *P* value is indicated above the bar graphs. (**D**) Endurance capacity of line 2 male mice measured as time to exhaustion during a treadmill running protocol (left panel, $n \geq 9$), Data are mean ± SEM; $n \geq 9$ mice per group. $^*P = 0.0326$ by unpaired Student's *t* test. Time to exhaustion for indicated female in line 1 (middle panel, $n \geq 5$, no difference between the genotype by unpaired Student's *t* test) and line 2 (right panel, $n \geq 5$, $^*P = 0.0293$ by unpaired Student's *t* test). Data are mean ± SD. Gene expression analysis in sedentary and run mice subjected to a moderate-intensity treadmill running protocol till near exhaustion. Muscles were harvested immediately after exercise. RT-qPCR showing mRNA levels of genes involved in glycolysis (**E**), glucose and fatty acid transport (**F**), and FA metabolism (**G**, **H**) relative to *Rpl30* in indicated mice. Data are mean ± SEM; $n = 4$. ($^*P < 0.05$, $^{**}P < 0.01$, two-way ANOVA). Selected *P* values are indicated above the bar graphs. Source data are available online for this figure.

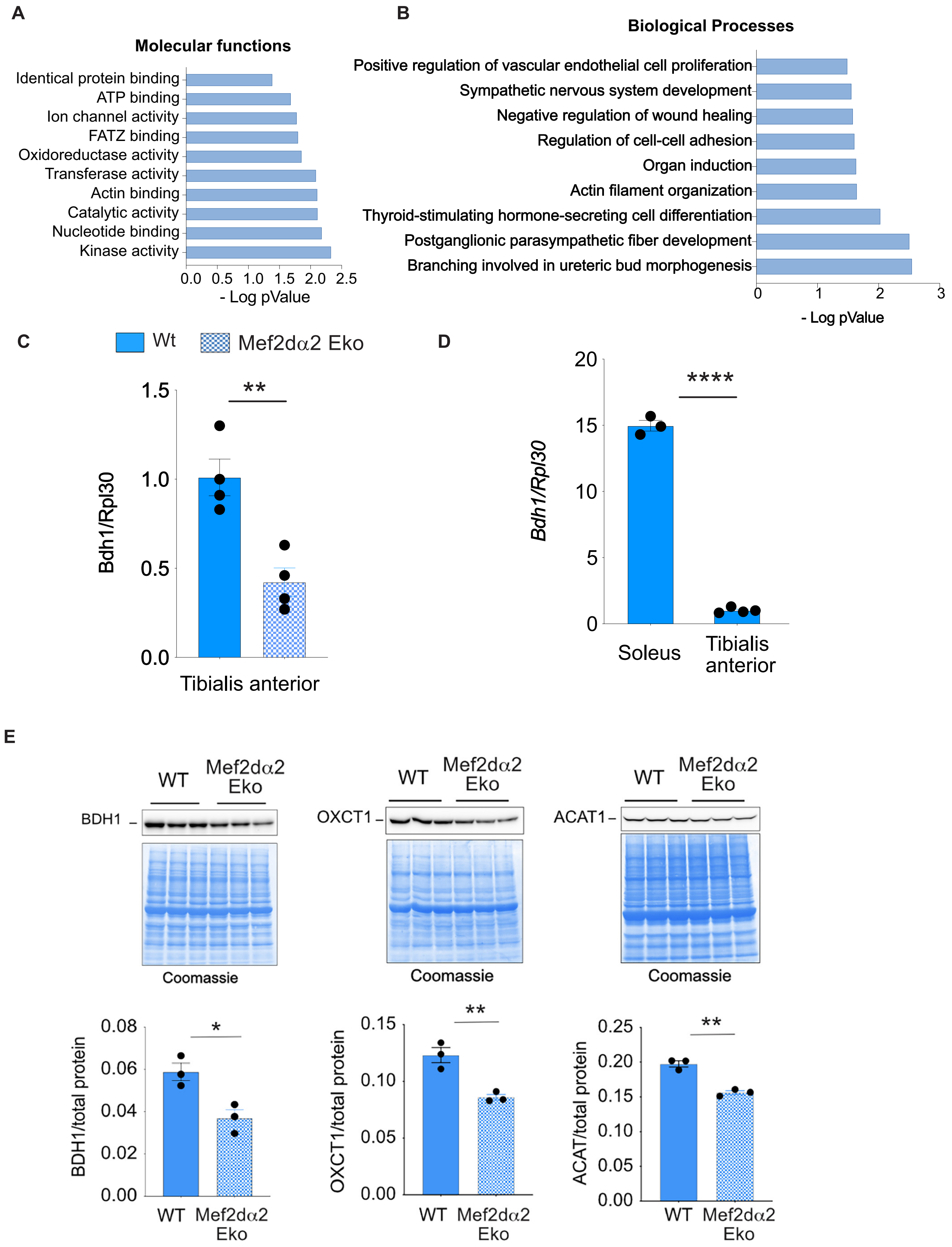
A
Molecular functions
Identical protein binding
ATP binding
Ion channel activity
FATZ binding
Oxidoreductase activity
Transferase activity
Actin binding
Catalytic activity
Nucleotide binding
Kinase activity
0.0 0.5 1.0 1.5 2.0 2.5
- Log pValue
B
Biological Processes
Positive regulation of vascular endothelial cell proliferation
Sympathetic nervous system development
Negative regulation of wound healing
Regulation of cell-cell adhesion
Organ induction
Actin filament organization
Thyroid-stimulating hormone-secreting cell differentiation
Postganglionic parasympathetic fiber development
Branching involved in ureteric bud morphogenesis
0 1 2 3
- Log pValue
C
Wt
Mef2dα2 Eko
Bdh1/Rpl30
**
Tibialis anterior
D
Bdh1/Rpl30
****
Soleus
Tibialis anterior
E
WT
Mef2dα2 Eko
BDH1
OXCT1
ACAT1
Coomassie
BDH1/total protein
OXCT1/total protein
ACAT/total protein
*
**
**

 © The Author(s)

Figure EV4. Mef2dα2 Eko mice show reduced muscle expression of ketolytic enzymes.

Gene ontology analysis using DAVID showing enrichment of molecular functions (**A**) and biological processes (**B**) among genes that are downregulated in MEF2Dα2 Eko muscles with *P* value < 0.05 using Fisher's exact test. (**C**) RT-qPCR showing relative expression of *Bdh1* normalized to *Rpl30* transcript levels in WT and MEF2Dα2 Eko TA muscles from line 1. Data are mean ± SEM; $n = 4$. $^{**}P < 0.01$ ($P = 0.004$ by unpaired Student's *t* test). (**D**) Relative expression of *Bdh1* transcripts normalized to *Rpl30* in indicated muscles from WT mice. Data are mean ± SEM, $n = 3$, $^{****}P < 0.0001$, ($P = 0.00000021$ by unpaired Student's *t* test). (**E**) Western blot showing BDH1, OXCT1, and ACAT1 level in soleus muscles of indicated mice from line 3. The panels below show BDH1, OXCT1, and ACAT1 level when normalized to total protein loaded as estimated by Coomassie staining of the same blot. Data are mean ± SEM, $n = 3$, $^{*}P < 0.05$ ($P = 0.0186$ for BDH1), $^{**}P < 0.01$ ($P = 0.0065$ for OXCT1, and $P = 0.0013$ for ACAT1 by unpaired Student's *t* test). Source data are available online for this figure.

© The Author(s)

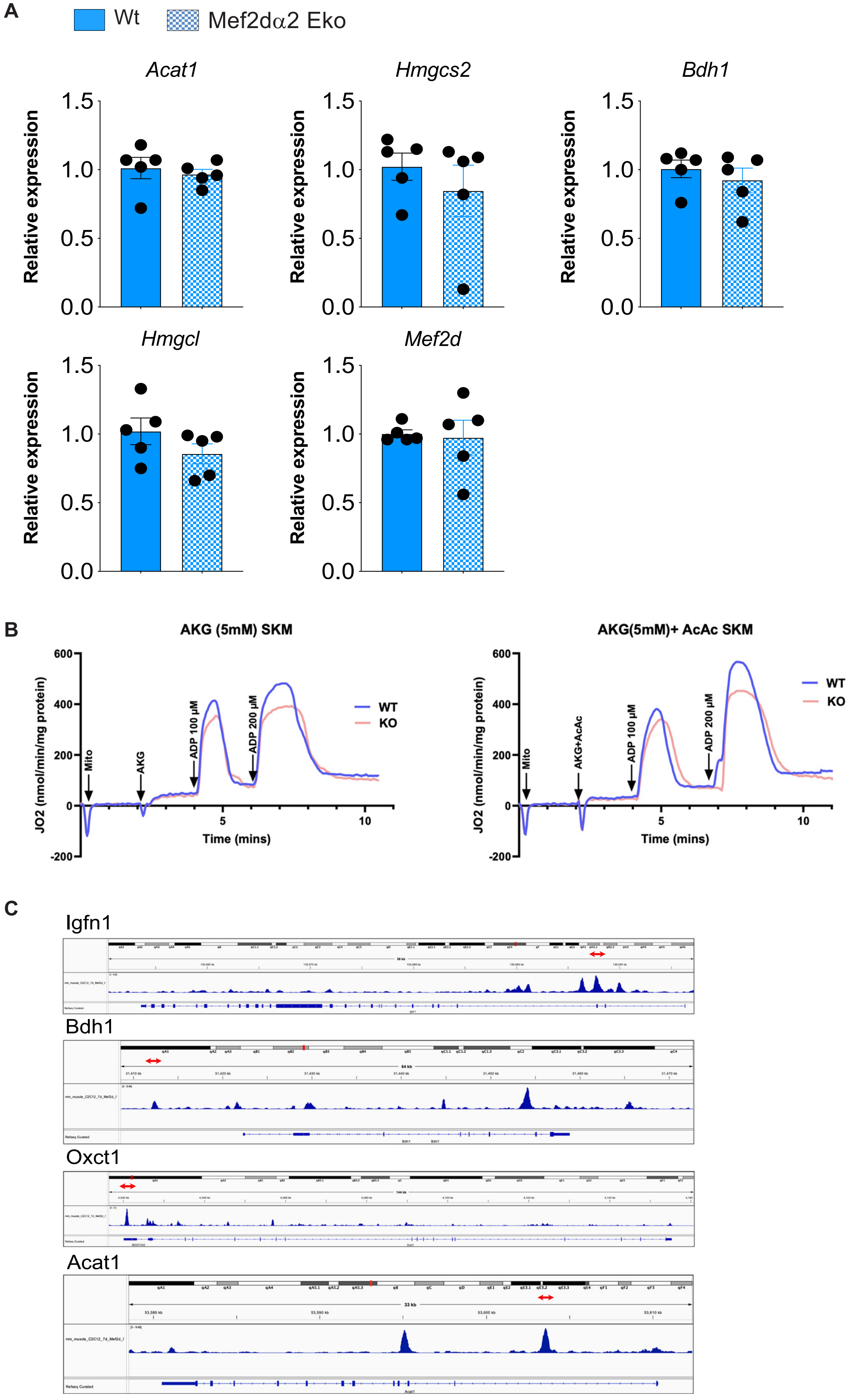
A
Wt
Mef2dα2 Eko
Acat1
Hmgcs2
Bdh1
Hmgcl
Mef2d
Relative expression
1.5
1.0
0.5
0.0
B
AKG (5mM) SKM
AKG(5mM)+ AcAc SKM
JO2 (nmol/min/mg protein)
Time (mins)
Mito
AKG
ADP 100 μM
ADP 200 μM
AKG+AcAc
WT
KO
600
400
200
0
-200
5
10
C
Igfn1
Bdh1
Oxct1
Acat1

Figure EV5. Gene expression related to ketone body metabolism does not differ in the livers of Mef2dα2 Eko mice.

(**A**) RT-qPCR showing relative expression of indicated transcripts normalized to *Hprt* transcript levels using total RNA from WT and MEF2Dα2 Eko mice livers. Data are mean ± SEM, $n = 5$, Student's *t* test found no differences in genotypes. (**B**) Representative time-courses of isolated mitochondrial respiration for WT and MEF2Dα2 Eko mice transitioning from state 1 to state 4 respiration under AKG± AcAc. The respiratory rates are expressed as nmol/min/mg mitochondrial protein. The transitions from state 1 to state 4 respiration were monitored by first adding isolated mitochondria (0.05 mg/mL) to the respiration buffer at $t = 0$ min leading to state 1. At $t = 2$ min, substrates were added to energize the mitochondria, which led to state 2 respiration. This was followed by sequential additions of incremental ADP concentrations (100 and 200 µM). AKG: Alpha-ketoglutarate and AcAc: acetoacetate. (**C**) MEF2D ChIP data from Gönczi et al viewed on IGV viewer (version 2.19.4) for Igfn1, Bdh1, Oxct1, and Acat1 gene locus. The bi-directional arrowed line shows the region where we designed our primers for our analyses. Source data are available online for this figure.

© The Author(s)
